# Electrically Insulating Rigid Multi-Channel Electrolyte Container for Customizable Electron Transfer in Zn-Halogen Batteries

**DOI:** 10.1007/s40820-025-02007-5

**Published:** 2026-01-05

**Authors:** Yifan Zhou, Yicai Pan, Yongqiang Yang, Taghreed F. Altamimi, Yunpeng Zhong, Dalal A. Alshammari, Zeinhom M. El-Bahy, Shuquan Liang, Jiang Zhou, Xinxin Cao

**Affiliations:** 1 School of Materials Science and Engineering, Key Laboratory of Electronic Packaging and Advanced Functional Materials of Hunan Province, Central South University, Changsha, 410083 People’s Republic of China; 2 Department of Materials Science and Engineering and Center of Super-Diamond and Advanced Films (COSDAF), City University of Hong Kong, Hong Kong SAR, 999077 People’s Republic of China; 3 Department of Applied Biology and Chemical Technology, The Hong Kong Polytechnic University, Hong Kong SAR, 999077 People’s Republic of China; 4 Department of Physics, College of Science, University of Hail, P.O. Box 2440, Hail, Saudi Arabia; 5 Christopher Ingold Laboratory, Department of Chemistry, University College London, London, WC1H0AJ UK; 6 Department of Chemistry, College of Science, University of Hafr Al Batin, P.O. Box 39524, Hafr Al Batin, Saudi Arabia; 7 Department of Chemistry, Faculty of Science, Al-Azhar University, Nasr City, 11884 Cairo Egypt

**Keywords:** Electrolyte container, Interfacial reaction regulation, Customizable electron transfer, Zn-halogen batteries

## Abstract

Recent advancements in Zn-halogen batteries have focused on enhancing the adsorptive or catalytic capability of host materials and stabilizing complex intermediates with electrolyte additives, while the halogen-ion electrolyte modifications exhibit strong potential for integrated interfacial regulation. Herein, we design an electrically insulating rigid electrolyte container to immobilize a liquid halogen-ion electrolyte for separator-free Zn-halogen batteries with customizable electron transfer. Robust hydrogen bonding of hydroxyl groups in SiO_2_ with fluorinated moieties in PVDF-*hfp* regulates Zn^2+^ solvation and suppresses H_2_O activity, while multi-channels formed by microcracks and interparticle gaps not only enhance mass transfer but also buffer interfacial electric field, jointly enabling a durable Zn plating/stripping. Effective confinement of intermediates also ensures the high reversibility across single-(I^−^/I^0^), double-(I^−^/I^0^/I⁺), and triple-(I^−^/I^0^/I⁺, Cl^−^/Cl^0^) electron transfer mechanisms at cathode, as evidenced by the double-electron transfer systems exhibiting a low capacity decay rate of 0.02‰ over 4500 cycles at 10 mA cm^−2^ and a high areal capacity of 11.9 mAh cm^−2^ at 2 mA cm^−2^. This work presents a novel “container engineering” approach to halogen-ion electrolyte design and provides fundamental insights into the relationships between redox reversibility and reaction kinetics.

**Supplementary Information:**

The online version contains supplementary material available at 10.1007/s40820-025-02007-5.

## Highlights

- A rigid electrolyte container (SiO_2_@PVDF-*hfp*) was designed to immobilize the liquid halogen-ion electrolyte, enabling separator-free Zn-halogen batteries.
- The container regulates Zn^2+^ solvation *via* hydrogen bonding regulation, while providing multi-channel structure for enhanced mass transfer, jointly enabling durable Zn plating/stripping.
- Effective confinement of intermediates ensures high reversibility across multi-electron transfer mechanisms, achieving an exceptionally low-capacity decay of 0.02‰ over 4500 cycles and a high areal capacity of 11.9 mAh cm^−2^.

**Supplementary Information:**

The online version contains supplementary material available at 10.1007/s40820-025-02007-5.

## Graphical abstract

**Figure:**
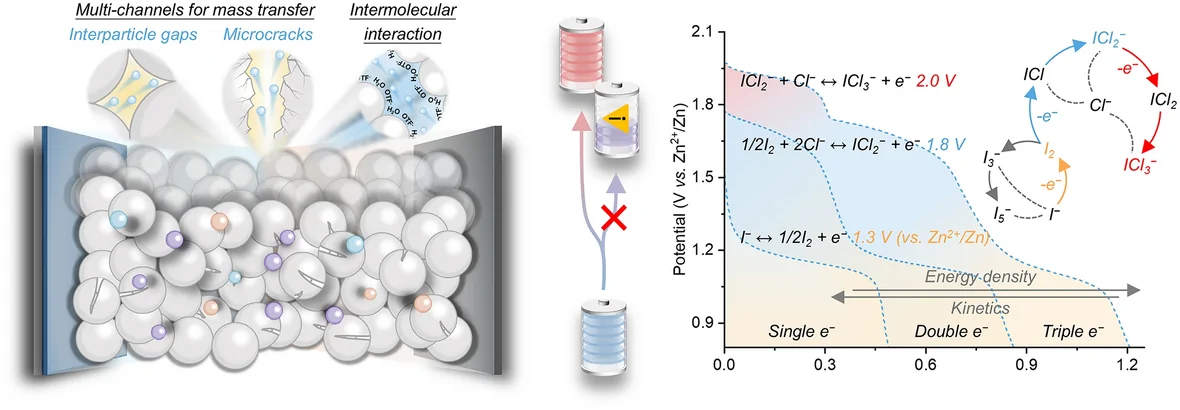

## Introduction

The past decade has witnessed the explosive growth in Zn-based batteries and their potential for grid-scale energy storage [1]. Distinct from rocking-chair systems driven solely by Zn^2+^ transport, the Zn-halogen batteries operate on a dual-ion mechanisms involving independent interfacial reactions (i.e*.*, Zn plating/stripping at anode and halogen redox at cathode) [2]. This configuration offers fast kinetics, high-energy efficiency, and design flexibility, yet the uncontrolled loss of active halogen species leads to short lifespan, thereby limiting their practical applications [3].

Halogens are incorporated into Zn-based batteries primarily through two strategies, including cathode blending and electrolyte additives [4]. The former is more commonly employed for higher utilization of charge carriers, while the latter streamlines the process by focusing optimization on the electrolyte itself [5]. Regarding physicochemical properties, halogen species such as I^−^ and Cl^−^ can form highly polar and unstable interhalogen compounds [6]. For instance, in a common double-electron system, I^−^ can complex with I_2_ to form I_3_^−^, which impairs kinetics. Meanwhile, the oxidized ICl species is prone to decomposition, causing the collapse of high-voltage plateau [7]. Hence, designing a system that can effectively confine these reactive species to enable reversible, high utilization redox remains a central goal.

To address these issues, various modification strategies have been proposed. A wide range of carbon substrates, doped frameworks, and metal compound composites were introduced into the cathode to enhance halogen confinement through physical adsorption or chemical anchoring, while catalytic components such as heteroatom dopants and transition metal sites were incorporated to accelerate redox kinetics [8–10]. Additionally, surfactants, organic chelators and pH-responsive polymers were added into electrolyte to stabilize reactive interhalogens via active association or pH regulation, so as to limit their migration [11–13]. These additives also modulated Zn^2+^ solvation and reduced H_2_O activity, contributing to inhibited gas evolution and uniform Zn deposition [14–17]. Also, functional separator coatings with ion-selective or adsorption capabilities, such as cation exchange polymers, metal–organic sieves, and Janus structures, were developed to restrict halogen anion migration and prevent species crossover [18]. From another perspective, while strategies often focus on regulating liquid electrolytes within passive glass fiber membranes, a promising alternative is to view the electrolyte and membrane as an integrated functional system. Holistically engineering this entity with synergistic interfaces, chemical constraints and tailored mechanical properties will create a new pathway toward developing high-performance Zn-halogen batteries suitable for diverse applications.

In this work, we move beyond conventional passive filled membranes or *in* situ polymer hydrogels to introduce a new “container engineering” strategy. We fabricate a pre-formed, rigid, and electrically insulating container composed of SiO_2_ and PVDF-*hfp* (denoted as SP), engineered not merely to host the liquid electrolyte (LE) but to actively regulate the electrochemical environment through integrated functions. The obtained rigid film (SPE) enables separator-free Zn-halogen batteries with improved reversibility and lifespan under customizable electron transfer mechanisms. The microcracks and interparticle gaps in SP form multi-channels for ion dissociation, storage, and migration, endowing SPE with ideal mass transfer capability. Meanwhile, strong hydrogen bonding between hydroxyl groups in SiO_2_ and fluorinated moieties in PVDF-*hfp* disrupts the original H_2_O network in LE, thus driving out H_2_O molecules from Zn^2+^ solvation and suppressing their activity. Coupled with buffering effect of multi-channels on ion concentration gradient and electric field, SPE promotes more uniform Zn plating and extended lifespan at a low density of 0.5 mA cm^−2^. Importantly, effective confinement of intermediates localizes redox reactions at cathode interface, achieving high reversibility across the single-(I^−^/I_0_), double-(I^−^/I_0_/I^+^), and triple-(I^−^/I_0_/I^+^, Cl^−^/Cl_0_) electron transfer mechanisms. This work presents a new strategy for electrolyte design in Zn-halogen batteries from the perspective of “container engineering” and provides fundamental insights into the correlation between redox reversibility and interfacial reaction kinetics.

## Experimental Section

### Materials

All reagents were purchased from Aladdin unless otherwise specified.

### Preparations of SP, SPE, and Zn-I Cells

#### ***Preparation of SiO***_***2***_***@PVDF-hfp Disk (Marked as SP)***

Firstly, fumed silica (SiO_2_) and poly(vinylidene fluoride-co-hexafluoropropylene) (PVDF-*hfp*) were mixed in a 4:1 weight ratio in N-methyl pyrrolidone (NMP) and stirred vigorously overnight. The mixture was dried at 60 °C to yield the initial SiO_2_@PVDF-*hfp* powder. Then, 100 mg of this powder was pressed at 10 MPa for 5 min using a stainless steel mold to form a SiO_2_@PVDF-*hfp* disk with a diameter of 16 mm. Finally, these disks were further vacuum-dried at 100 °C for 12 h to remove any residual solvent.

#### ***Preparation of Aqueous Electrolyte and Electrolyte-absorbing SiO***_***2***_***@PVDF-hfp Disk (Marked as SPE)***

The aqueous electrolyte was prepared by adding 3 M Zn(CF_3_SO_3_)_2_, 0.5 M potassium iodide (KI), and 2 M potassium chloride (KCl) to water and stirring until the solution became completely clear, among which KI serves as the sole iodine source in this research. The aqueous electrolyte, in its liquid form, is denoted as LE for subsequent comparisons. After immersing the dried SP disk in the aqueous electrolyte for overnight, a transparent SPE disk was obtained, which had absorbed approximately 70 μL of LE according to the calculation based on thermogravimetric results (Fig. S6).

#### *Preparation of Electrodes and Fabrication of Zn-I Cells*

Zn foil (50 μm) without any further modification was used as anode. For the cathode, a slurry was prepared by mixing activated carbon (YP-50F, Kuraray Co., Ltd), Ketjen Black, and polyvinylidene fluoride (PVDF) at an 8:1:1 weight ratio in NMP. The slurry was then uniformly cast onto the current collectors. The approximate activated carbon loading was ~ 2 mg cm^−2^ when using hydrophilic carbon fiber cloth (0.6 × 0.6 cm^2^ in coin cell, W0S1011, Ce Tech Co., Ltd.) and ~ 10 mg cm^−2^ for cathodes prepared with carbon felt.

CR2025 coin cells assembled in an open air at room temperature were used as battery system. The LE with soaking amount of 100 µL was loaded onto a glass fiber separator (GF/D, Whatman), whereas the SPE was used directly without any separator.

In addition, various cells were employed for characterization under different conditions, *e.g.*, Zn/Zn, Zn/Cu, stainless steel/stainless steel cells, pouch cell…

### Material Characterizations

X-ray diffractometer (Rigaku Mini Flex 600, Cu K*α* radiation, *λ* = 1.5418 Å) was used to determine the crystalline structure and phase composition. Morphology was characterized by a scanning electron microscope (SEM, TESCAN Mira 3), transmission electron microscope (TEM), and high-resolution TEM (HRTEM, FEI Titan G2, 60–300 kV) equipped with an energy-dispersive spectrometer (EDS). Elemental valence states were detected by an X-ray photoelectron spectroscopy (XPS, VG Escalab-250xi). Raman spectra were investigated using Lab RAM HR800, Fourier transform infrared (FT-IR) and UV–vis adsorption spectra were measured by Thermo Fisher Nicolet 6700 and Evolution 220. N₂ adsorption/desorption isotherms and pore size distributions were determined via a Brunauer–Emmett–Teller analyzer (BET, Micromeritics ASAP 2020). Thermogravimetric (TG) analysis was measured by NETZSCH STA 449C. Mercury intrusion porosimetry (MIP, Micro Active Auto Pore V 9600) was carried out to evaluate the pore size distributions ranging from 780 μm to 5 nm. Hardness and elastic modulus were measured on Bruker Hysitron TI 980. Differential electrochemical mass spectrometry was collected on Hiden HPR-20 OEMS.

### Electrochemical Measurements

All electrochemical performances were conducted on at least five independent cells for each condition to ensure reproducibility. The galvanostatic charge/discharge and Zn plating/stripping measurements were evaluated on a multi-channel battery test system (LAND CT2001A). Cyclic voltammetry (CV), chronoamperometry (CA), Tafel, linear sweep voltammetry (LSV), and electrochemical impedance spectroscopy (EIS) from 100 kHz to 10 mHz were conducted by an electrochemical workstation (CHI660E).

### Molecular Dynamics Simulations

Molecular dynamics (MD) simulations were conducted using the Forcite module in Materials Studio 2023 (Accelrys Inc.). Two models were constructed for comparison, with detailed molecular and ionic compositions summarized in Table 1. All simulations employed the COMPASS III force field. A time step of 1 fs was used. Each system was first equilibrated under the isothermal–isobaric (NPT) ensemble at 300 K and 0.1 GPa by a Berendsen barostat for 2 ns, followed by a 5 ns production run under the canonical (NVT) ensemble using a Nose thermostat. The simulation time was long enough to reach the equilibrium states of the systems. Then, radial distribution functions (RDFs) and hydrogen bonding information were calculated using the VMD analysis tool.

**Table 1:** Molecule numbers in MD simulations (The unit component of PVDF-*hfp* is set as (−CH_2_CF_2_−)_4_[−CF_2_CF(CF_3_)−])

Electrolyte	Component					
	Zn(CF_3_SO_3_)_2_	KCl	KI	H_2_O	SiO_2_	PVDF-*hfp*
LE	618	412	103	6491	–	–
SPE	264	176	44	1846	930	108

### Density Functional Theory Calculations

Density functional theory (DFT) calculations were carried out using the Vienna Ab initio Simulation Package (VASP) [19]. The exchange–correlation interactions were treated with the generalized gradient approximation (GGA) using the Perdew–Burke–Ernzerhof (PBE) functional, chosen for its reliable balance of computational efficiency and accuracy for quasi-solid-state systems, in conjunction with the projector augmented wave (PAW) method [20]. A vacuum layer of 20.0 Å was applied along the *z*-axis to eliminate the interactions between periodic images. Four-layer slab models were constructed to simulate the adsorption surface, which is a simplified model intended to isolate the intrinsic thermodynamics of the redox reactions, with the bottom two layers fixed to preserve bulk characteristics. A plane-wave cutoff energy of 520 eV was used, and a *Γ*-centered 3 × 1 × 1 k-point mesh was adopted. The electronic self-consistent field (SCF) convergence threshold was set to 1 × 10^−5^ eV atom^−1^ during geometry optimization and tightened to 1 × 10^−7^ eV atom^−1^ in single-point energy calculations. Then, structural relaxations continued until the residual forces on all atoms were less than 0.02 eV Å^−1^. Van der Waals interactions were accounted for using the DFT-D4 correction scheme, a modern and accurate method for capturing the interactions critical to adsorption. Then, the Gibbs free energy (*ΔG*) associated with halogen redox was calculated using the following equation:

1 $$\Delta G = E_{gs} + E_{zp} {-}T\Delta S$$

where *E*_*gs*_ is the ground-state energy, *E*_*zp*_ is the zero-point energy, and *TΔS* represents the entropy term. Both *E*_*zp*_ and *ΔS* were derived from vibrational frequency analyses based on DFT calculations. This overall approach is a standard practice aimed at elucidating qualitative reaction trends rather than replicating the full, complex solvated environment.

### COMSOL Simulations

The electric potential and current density distribution during Zn plating were simulated by the Tertiary Current Distribution in COMSOL Multiphysics. The electrochemical reaction kinetics at the electrode surface was described by the Butler–Volmer equation. Crucially, to simulate morphological evolution of the plated layer, Deforming Geometry interface was employed, where the boundary velocity of the electrode surface was set to be proportional to the local current density. The initial geometry included a sinusoidal profile on the electrode surface to represent initial protrusions. The simulations were run for 1000 s to capture the long-term plating behavior, and the corresponding dendrite growth height was extracted for quantitative analysis.

## Results and Discussion

**Fig. 1:**
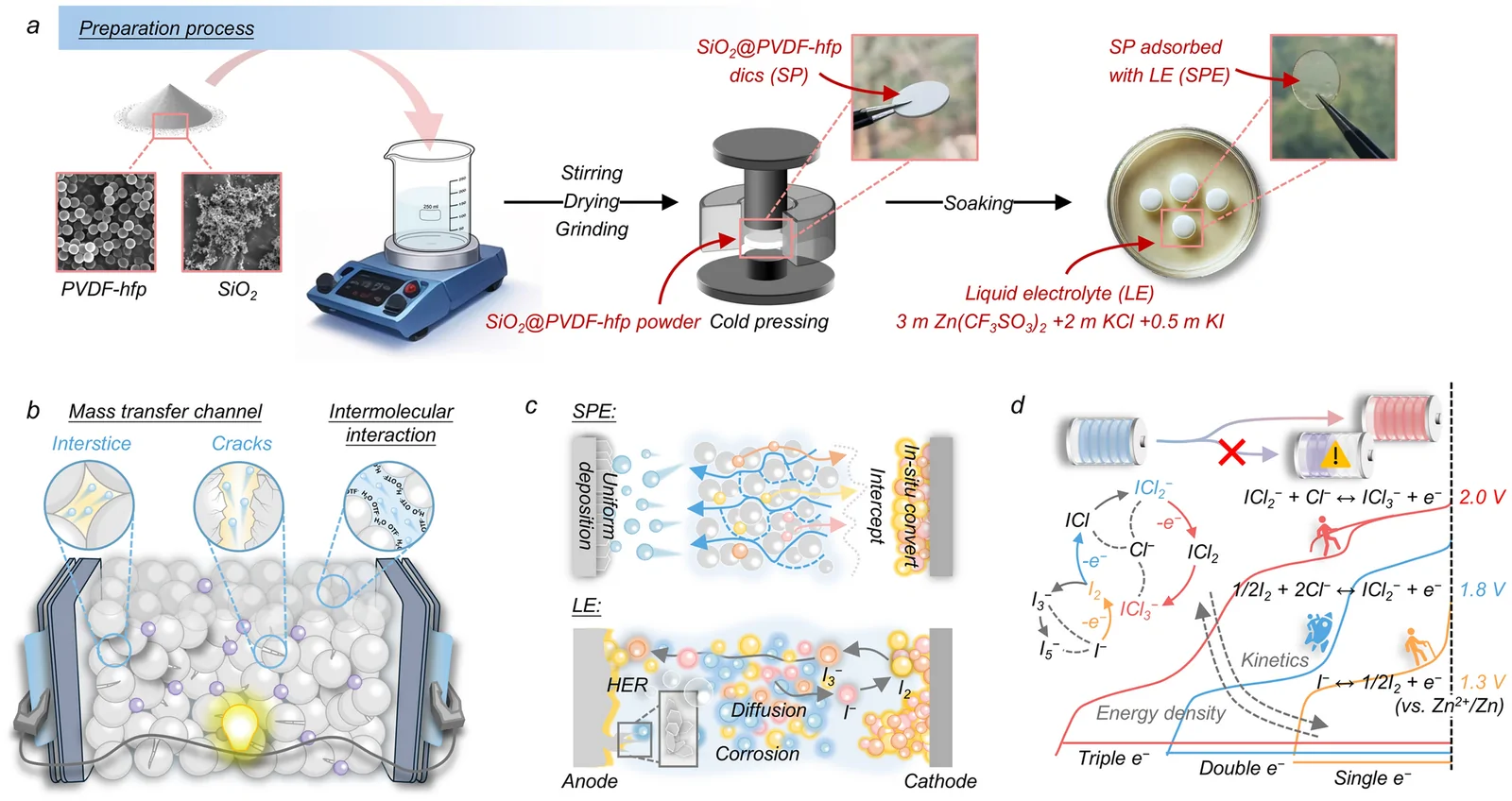
Schematic illustrations of SPE fabrication and functional mechanisms: **a** SPE preparation; **b** mass transport enhancement; **c** interfacial reaction regulation; and **d** customizable charge transfer Zn-halogen battery application

The electrolyte was prepared by fabricating rigid containers followed by a soaking process (Fig. 1a). First, fumed silica and poly(vinylidene fluoride-co-hexafluoropropylene) (PVDF-*hfp*) were vigorously mixed in an organic solvent and dried to form SiO_2_@PVDF-*hfp* powder, which was then pressed into disk shapes using a mold and further dried to produce mechanically robust SiO_2_@PVDF-*hfp* (denoted as SP, serving as the rigid container). These disks were subsequently immersed in a liquid electrolyte (denoted as LE for comparison) for soaking overnight, resulting in transparent electrolyte-adsorbed disks (denoted as SPE, representing the SP-host with electrolyte composite). The presence of potentially interconnected channels, interstices, and cracks in the internal architecture, along with possible interactions between PVDF-*hfp* chains and ions, enhances the ability of such a rigid container to provide storage sites and facilitate mass transfer pathways for the absorbed LE (Fig. 1b). Also, the incorporated framework within SPE helps expel water molecules and suppress their activity, offering advantages in controlling interfacial reactions such as gas evolution at the anode surface and shuttle effect of polyiodide species from the cathode surface (Fig. 1c). This approach provides a novel strategy for designing highly reversible and customized electron transfer Zn-halogen batteries with balanced energy density and kinetics (Fig. 1d).

### Physicochemical Evolution of Preparation

**Fig. 2:**
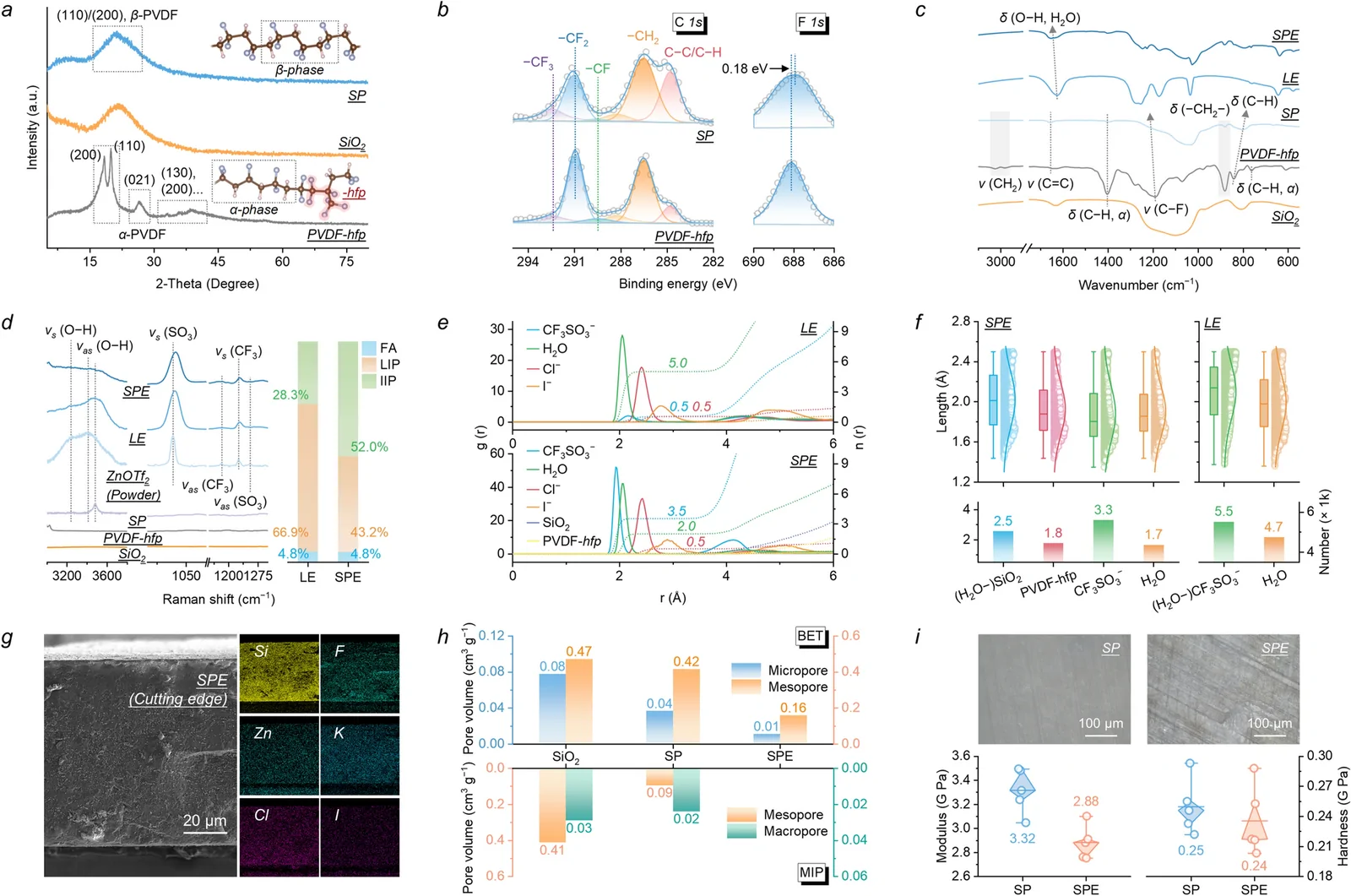
Physicochemical characterizations during the preparation: **a** XRD patterns of SiO_2_, PVDF-*hfp,* and SP; **b** XPS spectra of SP and PVDF-*hfp*; **c** FT-IR spectra of SiO_2_, PVDF-*hfp*, SP, LE, and SPE; **d** Raman spectra of SiO_2_, PVDF-*hfp*, SP, Zn(CF_3_SO_3_)_2_, LE, and SPE; **e** RDFs and **f** H-bonds statistics of LE and SPE derived from MD-simulated solvation boxes; **g** SEM images with EDS elemental mappings of SPE; **h** pore volume distribution of SiO_2_, SP, and SPE measured via BET and MIP; and **i** average hardness and elastic modulus of SP and SPE calculated from nanoindentation

The rigid containers (SP) were prepared by mixing SiO_2_ and PVDF-*hfp* in an N-methyl pyrrolidone (NMP) solvent, followed by drying and pressing, of which the XRD patterns are shown in Fig. 2a. The sharp diffraction peak of PVDF-*hfp* indicates its high crystallinity, with a doublet at 18.4°, 20.0°, and a peak at 26.6° corresponding to (020), (110), and (021) crystal planes of the monoclinic *α*-phase. Also, weak reflections at 33.2°, 35.9°, 38.8°, and 41.1° can be attributed to the (130), (200), (002), and (111) planes, respectively. In contrast, both fumed SiO_2_ and SP display only a broad peak around 20°, indicative of a highly amorphous structure. According to the crystal form of PVDF-*hfp*, this broad peak points to the (110), (200) planes of the orthorhombic *β*-phase, suggesting a phase transition during this process. In general, the *β*-phase with TTTT conformation in PVDF-*hfp* exhibits stronger polarity than the *α*-phase with TGTG' conformation, which is thought to enhance hydrogen-bonds (H-bonds) formation and ion dissociation (Fig. S1) [21, 22]. SiO_2_ exhibits hydrophilicity due to abundant hydroxyl groups on its surface, which likely form H-bonds (− OH···FC −) with PVDF-*hfp*. In this scenario, electron transfer (from F *δ*^−^ to H *δ*^+^) alters the binding energy between elements, as detected by X-ray photoelectron spectroscopy (XPS) [23]. The C 1*s* region after deconvolution by Gaussian fitting reveals the bonding information for –CF_3_, –CF_2_, –CF, –CO, –CH_2_, and C–H/C–C groups (Fig. 2b). Compared to PVDF-*hfp*, the peaks for –CF_3_, –CF_2_, and –CF in SP show a slight blue shift, together with C–F peak shifts by 0.18 eV toward the red in F 1*s* region, indicating the formation of H-bonds between the hydroxyl groups of SiO_2_ and fluorine groups of PVDF-*hfp* [24]. This is further supported by the enhanced intensity of Si–OH peak in Si 2*p* region, while the peak for Si^4+^ remains unchanged, indicating that SiO_2_ retains its original chemical state in SP (Fig. S2).

After immersing SP in LE overnight and drying, the SPE was obtained. The formation mechanism of this process was further investigated by Fourier transform infrared spectroscopy (FT-IR) and Raman spectroscopy. SiO_2_ exhibits characteristic peaks in FT-IR spectra, such as adsorption peaks at 807 and 1100 cm^−1^ corresponding to the symmetric stretching (*v*_*s*_) of Si–O and antisymmetric stretching (*v*_*as*_) of Si–O–Si, respectively. The peaks at 970 and 3440 cm^−1^ for binding vibration (*δ*) of Si–OH indicate the presence of hydroxyl groups again. Compared to PVDF-*hfp* and SiO_2_, the adsorption peaks decrease obviously in SP, in which the weakening of *v*(CH_2_) at 2980 cm^−1^ demonstrates the CH_2_ deprotonation in PVDF-*hfp*, while the appearance of *v*(C = C) at 1660 cm^−1^ and the blue shift of *v*(C–F) suggest the dehydrofluorination with mass reduction in PVDF-*hfp* chain [25]. Meanwhile, the red shift of *δ* (C–H) at 840 cm^−1^ and the almost vanished *δ*(C–H, *α*-phase) at 761 and 1403 cm^−1^ further prove the phase transition from *α*-phase to *β*-phase in the highly polar NMP solvent (Fig. 2c). Immediately after, compared to LE, SPE exhibits a reduced intensity of *v*(O–H) at 2950–3750 cm^−1^, a blue shift of *δ* (O–H, H_2_O), and a noticeable narrowing of Zn^2+^ solvation-related peak at 752–780 cm^−1^, reflecting that the introduction of SP container disrupts the strong H-bonds network in aqueous solution while enhancing the solvation of Zn^2+^ with anions (Fig. S3). The ion coordination was further analyzed by Raman spectra, as shown in Figs. 2d and S4. The *v*_*s*_(O–H) and *v*_*as*_(O–H) peaks at 3240 and 3408 cm^−1^ of LE disappear in SPE, leaving only a broad hump at 3483 cm^−1^ associated with structural water, which reveals the transition of water molecules from long-range order to an isolated state [26]. Additionally, the *v*(CF_3_) and *v*(SO_3_) at 764 and 1035 cm^−1^ in Zn(CF_3_SO_3_)_2_ shift to higher frequencies as the material transitions from powder to LE and then to SPE, suggesting enhanced coordination of CF_3_SO_3_^−^. By fitting the spectra in the 750–780 cm^−1^ region, Zn^2+^ association states are classified into free anions (FAs, 757 cm^−1^), loose ion pairs (LIP, 764 cm^−1^), and intimate ion pairs (IIPs, 768 cm^−1^) [27]. As results, the FAs fraction remains unchanged (4.8%) in LE and SPE, while the IIPs fraction enlarges from 28.3% to 52.0% in SPE, confirming stronger solvation of Zn^2+^ with CF_3_SO_3_^−^ in the SP container.

To explore the differences in solvation chemistry, molecular dynamics (MD) simulations were carried out on both LE and SPE. The 3D snapshots of the solvation boxes show a uniform distribution of all components, with no evident phase separation or molecular aggregation in the SPE composite (Fig. S5) [28]. Analysis of radial distribution function (RDF) reveals that the SP itself does not participate in the Zn^2+^ solvation shell. Instead, it enhances the interaction between Zn^2+^ and CF_3_SO_3_^−^ anions at the expense of displacing water molecules. This is evidenced by a shift in the average solvation cluster from [ZnCl_0.5_(CF_3_SO_3_)_0.5_(H_2_O)_5_]^+^ in the LE to the more anion-rich [ZnCl_0.5_(CF_3_SO_3_)_3.5_(H_2_O)_2_]^2−^ in the SPE (Fig. 2e) [29]. This restructuring is driven by changes in H-bonds network. In LE, the network is dominated by H_2_O–H_2_O (46%) and H_2_O–CF_3_SO_3_^−^ (54%) interactions. In SPE, these proportions decrease significantly, with the framework itself forming a substantial number of new H-bonds of H_2_O–SiO_2_ (27%) and H_2_O–PVDF-*hfp* (19%) (Fig. 2f; Table S1). Thermogravimetric (TG) analysis reveals a complex, two-stage absorption kinetic-related water molecule for the electrolyte (Fig. S6). A rapid initial uptake of loosely bound “physical water” is followed by a much slower formation of tightly bound “structural water,” which is associated with stable ion solvation sheaths. This reflects that SiO_2_ and PVDF-*hfp* in SP framework not only physically confine the water molecules but also actively restructure the H-bonds network over time. Notably, the clear water content fundamentally distinguishes SPE from solvent-free solid polymer electrolytes.

Physical structural evolution was examined using scanning and transmission electron microscopy (SEM, TEM). The morphology of fumed SiO_2_ consists of a loose network formed by fused nanoparticles approximately 10 nm in size, while PVDF-*hfp* exhibits uniform nanospheres with a diameter of ~ 300 nm. The SP container exhibits a compact and flat surface with a uniform elemental distribution. This structure is achieved as the phase transition in the NMP solution causes the PVDF-*hfp* spheres to collapse and then act as a binder under external pressure, forming a dense network of the SiO_2_ nanoparticles (Fig. S7). Furthermore, there are mesopores and macropores formed between SiO_2_ particles at the SP surface. Interstices ranging from nanometers to micrometers in length and fissures with widths approaching 500 nm were observed at both the edges and internal cross-sections, serving as multi-channels for transporting LE (Fig. S8). The SPE obtained after soaking appears transparent, and its morphology remains unchanged upon a heat treatment. Also, elemental mappings by energy-dispersive X-ray spectroscopy (EDS) of the surface, edge, and internal cross-section reflect the uniformity of ion distribution after LE penetration (Figs. 2g, S9). Inspired by these, Brunauer–Emmett–Teller (BET) test and mercury intrusion porosimetry (MIP) were carried out, as shown in Fig. 2h. The specific surface area of SiO_2_ decreases from 176.85 to 86.99 m^2^ g^−1^ after being mixed and pressed with PVDF-*hfp* (11.12 m^2^ g^−1^), while the pore volume reduces slightly from 0.47 to 0.42 m^3^ g^−1^, and the micropore distribution enlarges slightly. This is attributed to the closer contact between the nanoparticles under pressure as well as the formation of new micropores under collapse. Mesopores are formed in the cracks and gaps between the compact particles during this period, making the mesopores dominant in SP. While in the dried SPE, it shows a further decrease in both the specific area and pore volume, with the contents of micropores, macropores, and macropores unchanged significantly. This means that the LE infiltration does not damage the pore structure, and its crystallization can be extensively confined in SP container (Figs. S10, S11). In general, high specific area and narrow space built by mesopores are conducive for ion(s) adsorption and storage in SPE, while the ordered channels of macropores enhance the mass transfer [30, 31]. The nanoindentation test using Oliver–Pharr method was conducted to evaluate the mechanical properties [32]. The SP exhibits a hardness and elastic modulus of 0.25 and 3.32 GPa, respectively, which remained high at 0.24 and 2.88 GPa for SPE (Figs. 2i, S12). This retained high rigidity, coupled with ideal dimensional stability evidenced by a thickness swelling of less than 2%, suggests that SPE processes significant potential to mechanically inhibit Zn dendrites penetration (Fig. S13) [33].

Above all, the mechanically reinforced SPE demonstrates promising mass transfer characteristics. Chemically, the preserved H-bonds network between SiO_2_ hydroxyl groups and PVDF-*hfp* fluorinated moieties in SP container regulate Zn^2+^ solvation of introduced LE by disrupting H-bonds networks of water molecules as well as enhancing its interactions with CF_3_SO_3_^−^. Physically, the microcracks and interparticle voids within SiO_2_ matrix establish multi-channels, enabling efficient ion dissociation, temporary storage, and directional migration.

### Regulation of Anode Surface Reaction

**Fig. 3:**
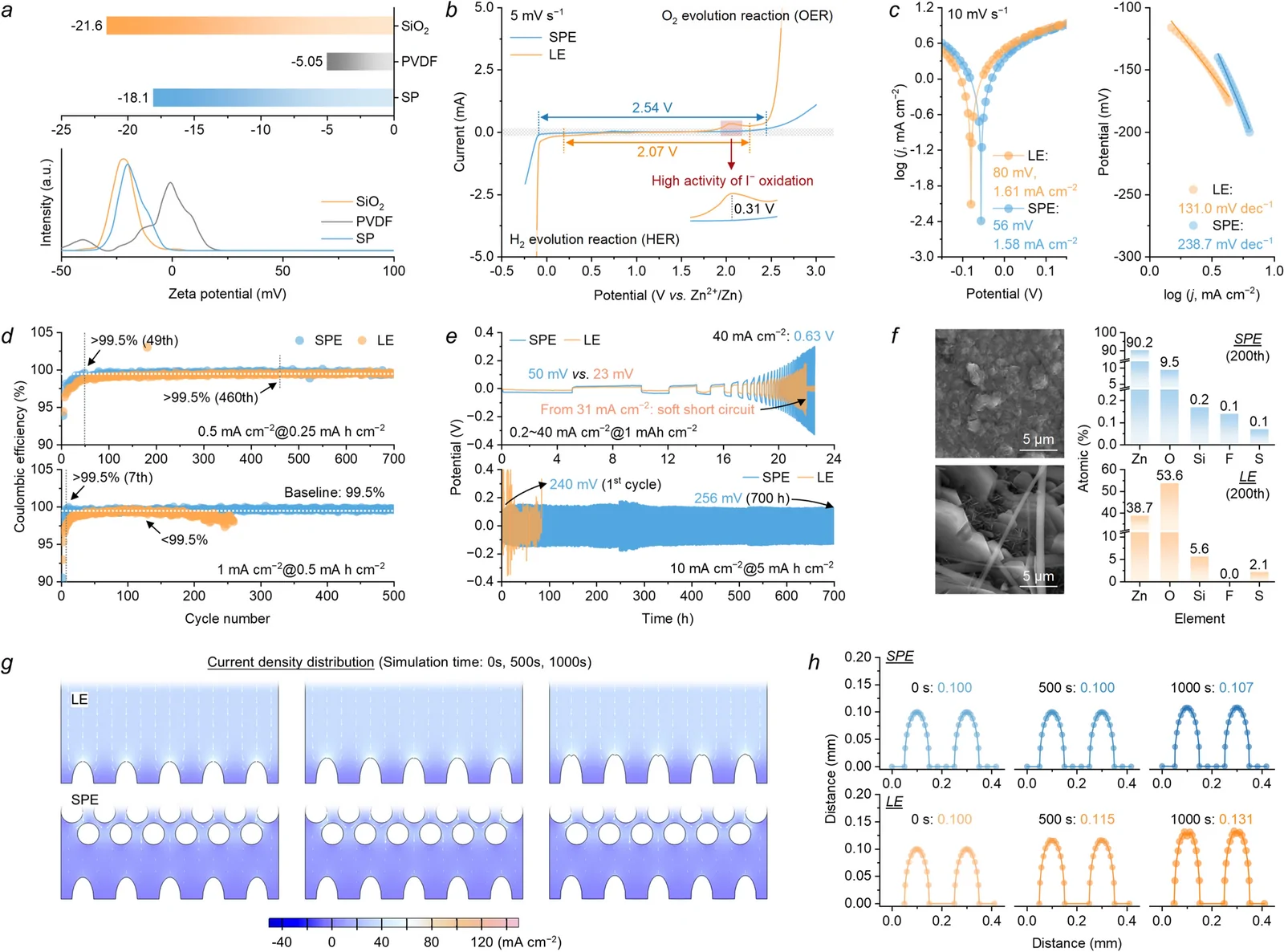
Investigation of anode surface reaction: **a** zeta potentials of SiO_2_, PVDF-*hfp,* and SP; **b** LSV curves at 5 mV s^−1^ of LE and SPE; **c** Tafel curves at 10 mV s^−1^ with Tafel slopes of LE and SPE; **d** CE plots at 0.5 mA cm^−2^@0.25 mAh cm^−2^ and 1 mA cm^−2^@0.5 mAh cm^−2^ in Zn/Cu cells with LE and SPE; **e** current density tolerance between 0.2 and 40 mA cm^−2^ to 1 mAh cm^−2^ and lifespan at 10 mA cm^−2^@5 mAh cm^−2^ of Zn-Zn cells with SPE and LE; **f** SEM images with EDS elemental ratios of deposited Zn anode after 200 cycles at 0.5mA cm^−2^@0.25 mAh cm^−2^ in LE and SPE; **g** current density distribution; and **h** dendrite height statistics over time in LE and SPE by COMSOL simulation

The zeta potential of SP and each component reflects their internal particle distribution and surface charge characteristics, where SP exhibits a slightly lower absolute *ζ* potential than SiO_2_ (− 18.1 *vs*. − 21.6 mV) while maintaining a high magnitude, indicating the robust electrostatic repulsion and good dispersibility of its particles. Combined with its electronegativity-driven cation capture capability, SP demonstrates potential advantages in optimizing interfacial contact and charge transfer efficiency (Fig. 3a) [34, 35]. This difference is further reflected in dielectric spectra, where SP exhibits a higher dielectric constant and lower dielectric loss than SiO_2_ in the low-frequency range, indicating its enhanced ionic dissociation and subsequent ion transport (Fig. S14) [36]. The Zn^2+^ transference number (${\mathrm{t}}_{{\mathrm{Zn}}^{2+}}$) and ionic conductivity (*σ*) of LE and SPE, measured in Zn/Zn and stainless steel/stainless steel cells for revealing the differences in mass transfer capabilities between liquid- and quasi-solid-state electrolytes, show *σ* values of 0.058 and 0.224 S cm^−1^ and ${\mathrm{t}}_{{\mathrm{Zn}}^{2+}}$ values of 0.58 and 0.70 in SPE and LE, respectively (Figs. S15, S16). The diminished ionic conductivity of SPE stems from the electrically insulating SiO_2_, yet its ion transport performance remains superior to most reported quasi-solid-state electrolytes owing to its internally rich H-bonds network and multi-channels formed between interconnected particles [37, 38]. Linear sweep voltammetry (LSV) at 5 mV s^−1^ reveals an extended electrochemical stability window for SPE (2.54 V) versus LE (2.07 V), with SPE showing a flattened slope at both ends, indicating suppressed gas evolution and eliminating redox peaks in the iodine conversion region (Fig. 3b). Such a phenomenon was further confirmed by Tafel curves at 10 mV s^−1^, in which SPE exhibits lower exchange current density (1.58 vs. 1.61 mA cm^−2^), higher equilibrium potential (56 vs. 80 mV), and larger Tafel slope (238.7 vs. 131.0 mV dec^−1^) than that in LE (Fig. 3c). This demonstrates that SPE effectively enhances the anti-corrosion capability on anode surface, especially in hydrogen evolution reaction (HER) suppression. This was unequivocally confirmed by *in* situ differential electrochemical mass spectrometry (DEMS), which quantifies a dramatically lower H_2_ evolution rate for SPE system compared to LE in symmetric cells (Fig. S17).

The Zn/Cu cells were assembled to investigate the improvement of Zn plating/stripping behavior, in which the SPE enabled a Coulombic efficiency (CE) of 99.5% within just 49 cycles at 0.5 mA cm^−2^, a milestone that required 460 cycles in LE (Fig. 3d). Such a performance was highly reproducible, as demonstrated by the excellent cell-to-cell consistency shown in Fig. S18. Even at 1 mA cm^−2^, the SPE maintained high reversibility, whereas the CE in LE-based cell deteriorated. While the SPE exhibited higher voltage hysteresis due to its moderate conductivity, this was a worthwhile trade-off for the dramatic improvement in stability and CE (Fig. S19). This advantage was further demonstrated in Zn/Zn cells under more demanding conditions. The SPE showed exceptional rate tolerance up to 40 mA cm^−2^, in stark contrast to the LE-based cell which suffered a “soft short-circuit” above 31 mA cm^−2^ (Fig. 3e). Furthermore, it achieves a remarkable lifespan of over 700 h at a demanding condition of 10 mA cm^−2^ to 5 mAh cm^−2^, whereas the cell with LE failed in the initial cycles (Fig. S20).

To understand the origin of this enhanced stability, the deposited morphology and composition on the anode were investigated. Post-mortem analyses were conducted on anodes cycled at a low current density of 0.5 mA cm^−2^, a condition where chemically-driven parasitic reactions such as gas escape and by-product formation are particularly prominent [39]. *Ex* situ SEM revealed a dense, flat morphology for the electrode from SPE, in contrast with the disordered flake stacking observed from LE (Figs. S21, S22). EDS mappings with quantitative analysis showed that the oxygen content on SPE-cycled anode was only 9.4%, far lower than 53.6% on LE-cycled anode (Fig. 3f). This enhanced chemical stability directly translates to a more uniform physical Zn plating, as visualized by *in* situ optical microscopy (Fig. S23). While distinct convex protrusions appeared within 5 min in LE and evolved into irregular “dendritic structures,” the electrode in SPE remained flat throughout the measurement, showing only a slight and uniform thickening. COMSOL simulations provide modeled current density distributions in free- and channel-confined mass transfer (Fig. 3g). Initial surface protrusions trigger a classic “tip effect” in LE system, leading to a severely focused current density with peak value at the tips reaching over 5 times the surrounding valley. This theoretical current focusing is then quantified by the derived dendrite height statistics, which show a rapid, accelerating growth rate characteristic of uncontrolled plating (Fig. 3h). Furthermore, these current “hot spots” are regions of high local overpotential that drive parasitic side reactions like HER, which is in good agreement with our Tafel analysis. *Ex* situ XRD patterns revealed that the electrodes from SPE maintained a pure Zn phase over 200 cycles, while LE counterparts exhibited additional hydroxides (Zn_5_(OH)_8_Cl_2_·H_2_O) and oxides (ZnO), due to the release of hydroxide ions from these side reactions (Fig. S24). In stark contrast, the multi-channel structure in SPE acts as an effective ion flux homogenizer, maintaining a remarkably uniform current density, which provides a clear theoretical explanation for the slow, suppressed dendrite growth and ultimately underpins the experimentally observed clean, by-product-free anode surface and the pure Zn phase in the XRD patterns.

The aforementioned results prove the advantages of SP in regulating the anode surface reaction. The electronegative network formed by dispersed SiO_2_ as well as disruption of the original H-bonds network of water in LE efficiently drives Zn^2+^ transport while suppressed gas evolution reaction, thereby enhancing the reversibility and stability. In addition, SPE with mechanical strength and multi-channels buffers electric fields and concentration gradients between interfaces, promoting uniform Zn plating.

### Application of SPE in Customizable Electron Transfer Zn-Halogen Batteries

**Fig. 4:**
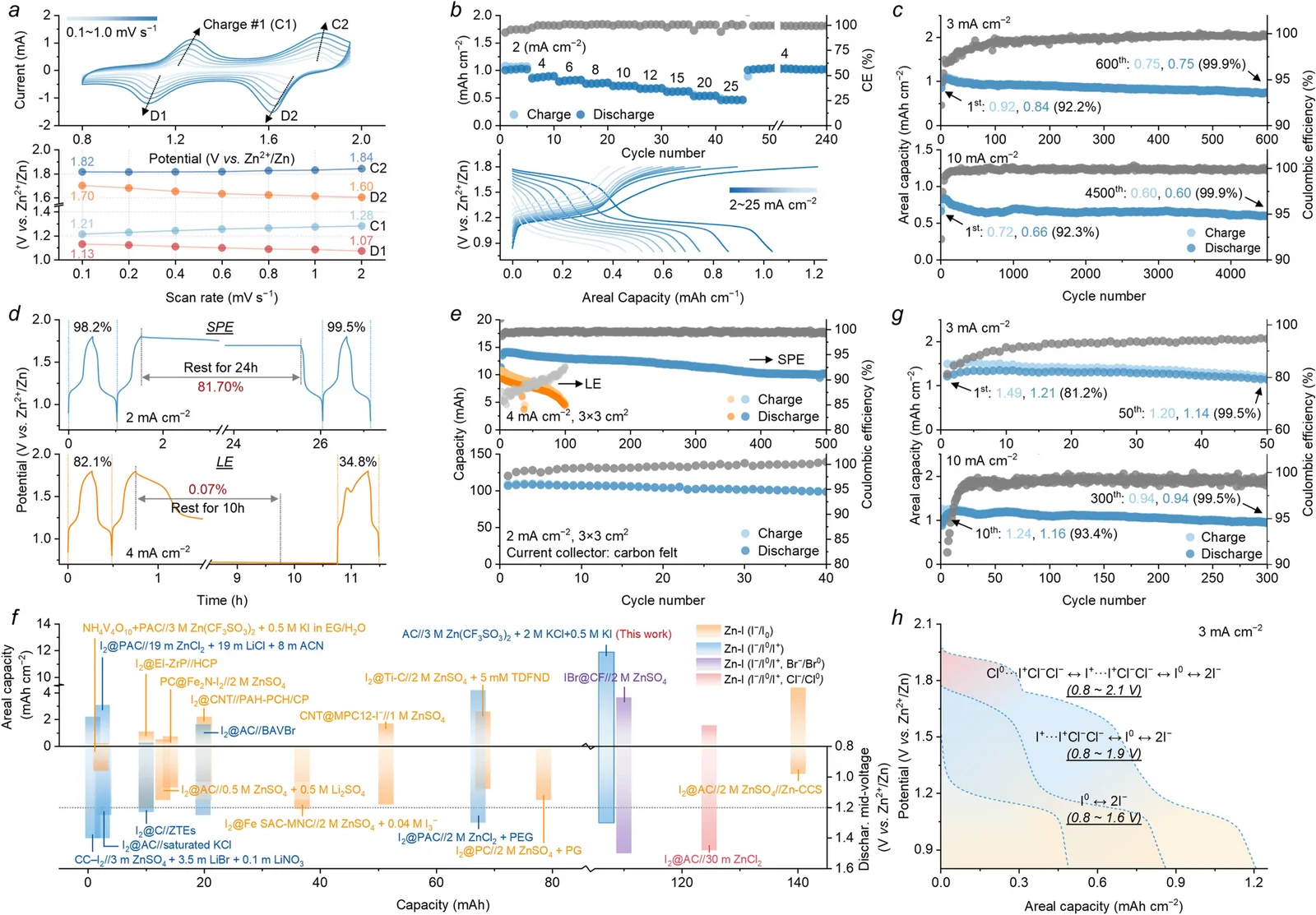
Electrochemical performances of SPE in customizable charge transfer Zn-halogen batteries: **a** CV curves with redox peak voltages at 0.1–1.0 mV s^−1^; **b** rate capability with voltage curves at 2–25 mA cm^−2^, **c** lifespans at 3 and 10 mA cm^−2^ and **d** resting tests (*vs*. LE) in coin cells with double-electron transfer; **e** lifespans using a carbon fiber cloth current collector at 4 mA cm^−2^ (*vs*. LE) and a carbon felt current collector at 2 mA cm^−2^ in pouch cells (3 × 3 cm^2^) with double-electron transfer; **f** comparisons of areal capacity, discharge mid-voltage, and capacity in other metal-halogen batteries; **g** lifespans at 3 and 10 mA cm^−2^ in coin cells with triple-electron transfer; and **h** summarized performances of SPE in customizable charge transfer Zn-halogen coin cells

Due to chloride-induced corrosion of stainless steel under electric fields, most reported Zn-halogen cells with multi-electron transfer rely on Swagelok-type devices [6, 7]. Since the strong ion adsorption with dissociation and suppressed water activity in SP, SPE holds potential as an electrolyte for multi-electron transfer Zn-halogen coin cells, with potassium chloride and potassium iodide in the soaking solution serving as the only driving forces of this reaction (Fig. S25). Cyclic voltammetry (CV) curves at scan rates of 0.1–1.0 mV s^−1^ were firstly collected within the voltage range from 0.80 to 1.95 V, revealing two pairs of redox peaks (1.21/1.13 V and 1.82/1.70 V at 0.1 mV s^−1^) corresponding to a double-electron transfer of I^−^ (I^−^  → I^0^ → I^+^) without any accompanying side reactions. Notably, these peaks shifted slightly as the scan rate increased, indicating rapid reaction kinetics at both interfaces (Fig. 4a). Fitting the peak current and scan rate yielded the *b*-values around 0.4–0.7 in these redox peaks, revealing a combined diffusion–capacitive-controlled behavior. While quantitative convolution showed capacitance gradually dominating the capacity contribution with increased scan rates (from 40% at 0.1 mV s^−1^ to 85% at 1 mV s^−1^), further confirming enhanced kinetics and efficient energy storage (Fig. S26).

In terms of the rate performance, SPE delivers average areal capacities of 1.02 mAh cm^−2^ at 2.0 mA cm^−2^ and 0.46 mAh cm^−2^ at 25 mA cm^−2^, with Coulombic efficiency (CE) improving from 95.5% to nearly 100% (Fig. 4b). Upon current density recovered to 4 mA cm^−2^, the cell operates stably for an additional 200 cycles. Regarding lifespan, capacity decay rates are only 1.8‰ over 600 cycles at 3 mA cm^−2^ and 0.02‰ over 4500 cycles at 10 mA cm^−2^, respectively, with a distinct plateau observed at high current density. A capacity fading primarily occurs in the first 100 cycles, during which an equilibrium interface forms concurrently, driving the CE to reach 99.5% (Figs. 4c, S27). Inevitably, the I^0^/I^+^ redox is inhibited by polarization at 20 mA cm^−2^, resulting in a single-electron transfer that inhibits highly reproducible stability, with no capacity decay and CE of nearly 100% over 4000 cycles (Fig. S28). This phenomenon reflects a design trade-off between ionic conductivity and interfacial reaction control. While SPE’s moderate conductivity inevitably increases polarization, its key advantage is enabling stable operation under demanding current densities. Resting tests from the fully charged state were conducted to assess the stability of oxidation products in LE and SPE, in which SPE maintains a CE of 81.7% after standing for 24 h, whereas LE drops sharply to 0.07% within just 10 h (Fig. 4d). Overall, SPE demonstrates superior redox kinetics as well as reaction reversibility and stability in coin devices. Furthermore, pouch cells (3 × 3 cm^2^) were assembled, in which SPE performs a discharge capacity of 10.3 mAh with a retention of 93% over 400 cycles at 4 mA cm^−2^. In contrast, LE shows a lower initial capacity of 9.3 mAh, poor CE (< 90%), and a significantly reduced retention of 46.2% over 100 cycles, indicating limited reversibility of iodine redox in aqueous electrolyte. To further enhance the areal capacity, the carbon fiber cloth current collector was replaced with carbon felt. Owing to its three-dimensional, non-woven architecture, the carbon felt supports a higher loading of activated carbon and provides a vastly expanded reaction interface for enhancing the reaction depth. As a result, a pouch cell (3 × 3 cm^2^) achieves a significantly improved capacity of 107 mAh, equivalent to an areal capacity of 11.9 mAh cm^−2^, while remaining stable for over 40 cycles at 2 mA cm^−2^ (Figs. 4e, S29). Compared to the reported Zn-halogen pouch cells, SPE demonstrates a clear advantage in areal capacity and exhibits a higher discharge mid-voltage among Zn-I pouch cells with double-electron transfer due to its reduced polarization (Fig. 4f; Table S4).

To evaluate the suitability of SPE in facilitating chloride redox (Cl^−^/Cl^0^), the charging cutoff voltage of coin devices was raised to 2.1 V. While operation at this high voltage raises concerns about stability, the process proceeds via a non-gas-evolving interhalogen pathway (i.e., from ICl_2_^−^ to ICl_3_^−^). This conclusion is clearly supported by in situ DEMS, which detected no Cl_2_ signals during high-voltage charging (Fig. S30). Additionally, post-mortem analysis confirms that SPE effectively mitigates the associated corrosion risk (Fig. S31) [40]. As a result, the cell delivers an initial areal capacity of 1.21 mAh cm^−2^ with a lifespan of 50 cycles at 3 mA cm^−2^. Even at 10 mA cm^−2^, a peak capacity of 1.16 mAh cm^−2^ with 81% retention was achieved over 300 cycles (Fig. 4g). The robustness of this long-term stability was validated through tests on multiple cells, which exhibited excellent consistency (Fig. S32). A distinct voltage plateau for Cl^−^/Cl^0^ redox observed at both current densities reveals that the excellent kinetics of SPE are maintained in a triple-electron transfer process (Fig. S33). Compared to reported metal-halogen coin cells, SPE-supported cells exhibit significant improvements in capacity, lifespan, and reversibility across multiple electron transfer mechanisms (Fig. S34). Additionally, a boarder comparison with optimized strategies involving Janus separators, hydrogels, and quasi-solid electrolytes for Zn-based batteries underscores the superior overall balance of properties in our system (Table S5). At this stage, the SPE with effective interfacial reaction control and optimized kinetics characteristics has promising potential as an electrolyte for Zn-halogen cells with customizable charge transfer (Fig. 4h).

### Reversible and Kinetic Optimizations of Cathode Surface Reaction

**Fig. 5:**
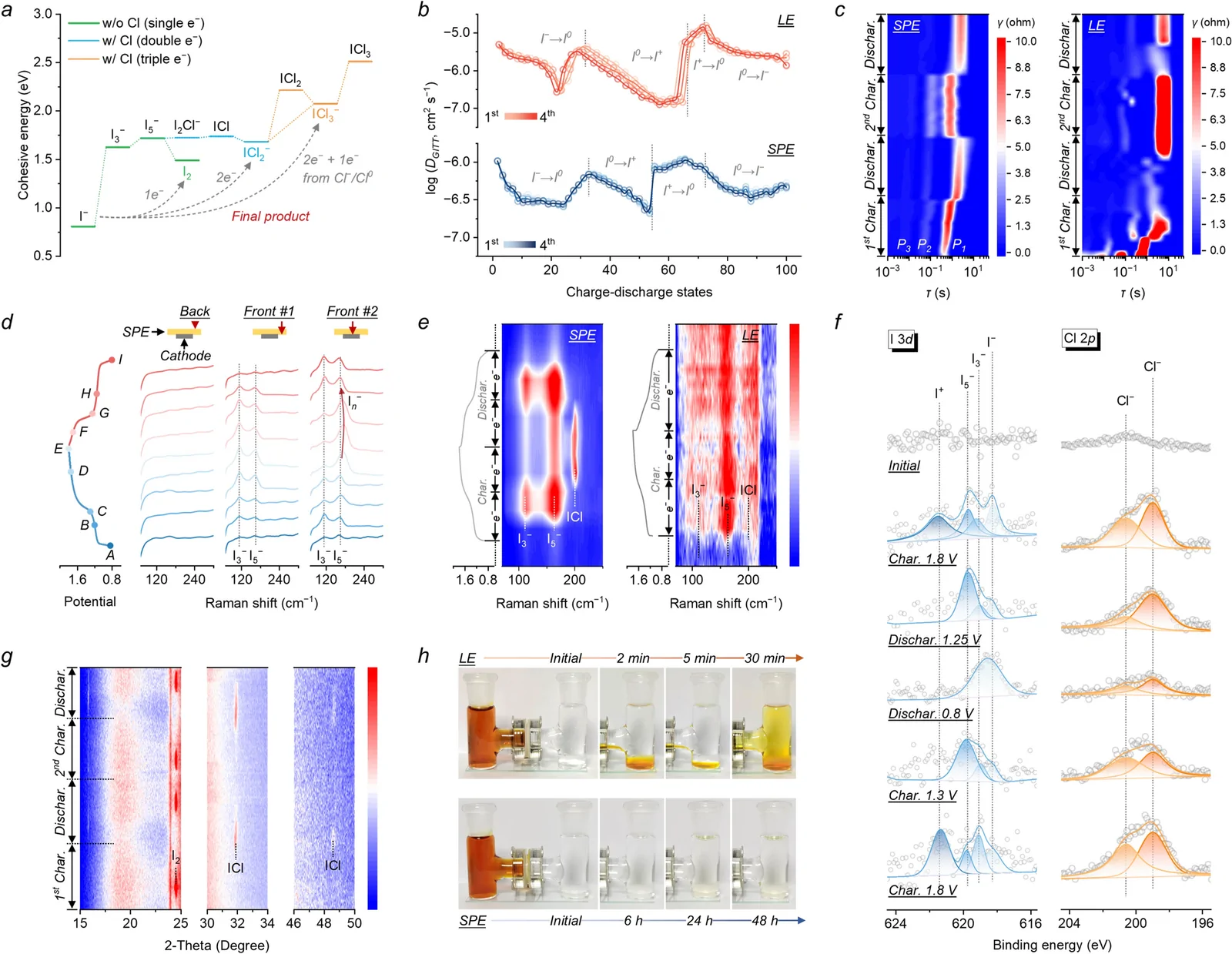
Reversibility and kinetics of cathode surface reaction: **a** Cohesive energies of interhalogen species involved in multi-electron transfer; **b** diffusion coefficients (*D*_*GITT*_) calculated from GITT at 3 mA cm^−2^ and **c** DRT spectra at timescales of 10^−3^ to 50 s derived from *ex* situ EIS measurements in full cell with LE and SPE; **d** *ex* situ Raman spectra of SPE at different positions in full cell; **e** *in* situ Raman spectra of cathode in full cell with LE and SPE; **f** *ex* situ XPS spectra and **g** *in* situ XRD patterns of cathode in full cell with SPE; and **h** optical images of polyiodide diffusion in SPE and LE within an H-type device

Generally, multi-electron transfer in iodine species can be activated and controlled by regulating the stability of interhalogen compounds [7]. Given the diversity of these inter-compounds, the pathways of halogen redox in this system were inferred by calculating the potential energies of possible products (Fig. 5a). For single-electron transfer, the complete oxidation product of I^−^ is I_2_, which exhibits lower energy compared to other polyiodide ions and limited solubility in aqueous solvent. In terms of double-electron transfer, forming ICl_2_^−^ is energetically more favorable than ICl, resulting in a greater tendency for I^+^ to coordinate with two Cl^−^ [41]. Subsequently, ICl_2_^−^ is further oxidized into ICl_2_ and ultimately stabilized as ICl_3_^−^, in which Cl^0^ is immobilized through chemical bonding in interhalogen compound rather than physical adsorption [40].

With the halogen redox pathway clarified, kinetic differences and interfacial control between SPE and LE were evaluated based on the double-electron transfer mechanism. Galvanostatic intermittent titration technique (GITT) measurement at 3 mA cm^−2^ was performed to reveal kinetic characteristics. Overall, the similar trends of *D*_GITT_ across different cycles reflect consistent kinetic behaviors in LE and SPE, with the *D*_GITT_ values in LE being approximately one order of magnitude higher than those in SPE owing to the intrinsic mass transfer advantages of aqueous solvent. Specifically, the *D*_GITT_ in LE exhibits a significant decline in both the I^−^/I^0^ and I^0^/I^+^ stages, where the former represents an energy barrier arising from the liquid–solid-phase transition, and the latter results from kinetics deterioration caused by excessive ICl formation [42]. The spontaneous reduction of highly reactive ICl also triggers a sharp increase in *D*_GITT_ upon the subsequent discharging. In contrast, SPE displays a similar trend but with notably reduced fluctuation, suggesting that SPE offers a buffering effect on kinetics behavior (Figs. 5b, S35).

To gain deeper insights, *ex* situ electrochemical impedance spectroscopy (EIS) was collected within the first two cycles. The bulk resistance (*R*_b_) in SPE shows a slight increase as charging, while charge transfer resistance (*R*_ct_) remains stable. No additional semicircles point to mass transfer between SPE and cathode, further supporting the fast kinetic behavior. In contrast, LE exhibited initially higher *R*_b_ that gradually decreases with cycling, accompanied by irregular data points indicative of complicated internal reactions and side products (Fig. S36). Thereafter, the represented distribution relaxation times (DRT) were calculated with the results showing three relaxation times (*τ*) in the scales of 10^−3^–10 s, which are assigned to ion transport within the electrolyte (*P*_3_), charge transfer during redox reactions of iodine species (*P*_2_), as well as mass transport of products at the interface and within the cathode (*P*_1_) [43–45]. The variations in *g*(*τ*) suggest that the overall kinetics in both SPE and LE are primarily influenced by *P*_1_ process. Specifically, SPE exhibits shorter timescales in *P*_1_ process, along with consistently lower *g*(*τ*) in *P*_2_ and *P*_3_ processes during charging, demonstrating accelerated iodine conversion at interface as well as rapid adsorption–desorption by the porous activated carbon, particularly during oxidation. In contrast, LE displays higher initial *g*(*τ*) across all three processes, in agreement with its elevated *R*_b_ and *R*_ct_ in EIS results, which subsequently decline rapidly following redox progression. Notably, *g*(*τ*) for *P*_1_ process significantly enlarges again during second charging, attributable to residual solid-phase products (e.g., I_2_) from incomplete reduction impeding subsequent mass transfer. Irregular timescale distributions of *P*_2_ and *P*_3_ further highlight the complex internal kinetics within LE (Fig. 5c).

Energy storage mechanism and reversibility were confirmed by monitoring iodine species evolution at different interfaces within the battery. Ex situ Raman spectra at various positions in SPE were carried out, in which typical scattering peaks of I_3_^−^ and I_5_^−^ are located at 113 and 164 cm^−1^, respectively (Fig. 5d). Throughout cycling, no iodine-related peaks are detected on the back side of SPE (adjacent to anode, marked as *Back*), whereas the front side away from cathode (*Front #1*) displays reversible I_3_^−^, I_5_^−^ signals with former dominating in intensity. This phenomenon is also observed on the front side aligned with cathode (*Front #2*), but here the I_5_^−^ signal exhibits higher intensity and a blue shift at high voltage, indicative of higher-order polyiodide ions (I_*n*_^−^) formation. These results suggest that SP framework effectively regulates the iodine concentration gradient, strictly confining polyiodide ions at the cathode surface, thus preventing interfacial crosstalk and charge carrier loss. Then, in situ Raman was used to investigate the differences in cathode between SPE and LE, in which the I_3_^−^ and I_5_^−^ signals, along with a ICl signals at 200 cm^−1^ sequentially appears and subsequently disappear in SPE, highlighting superior reversibility. In contrast, these signals persist continuously in LE, reflecting limited controllability (Fig. 5e).

*Ex* situ XPS spectra of cathodes were analyzed to clarify the evolution of chemical valence states. Initially, no peaks appeared in I 3*d* and Cl 2*p* regions. Upon charging to 1.8 V, a series of peaks emerge in I 3*d* region, which can be deconvoluted by Gaussian–Lorentzian fitting into sub-peaks representing I⁺, I_5_^−^, I_3_^−^, and I^−^, with the integrated area of I^+^ dominating. Concurrently, the Cl 2*p* region shows the emergence and intensification of distinct peaks corresponding to Cl^−^ upon charging to 1.8 V. This indicates that Cl^−^ does not participate directly in the redox reaction but instead migrates to and accumulates at the cathode interface. This accumulation of Cl^−^ is consistent with the formation of I^+^, providing the necessary counter-ions to form interhalogen complexes such as ICl_2_^−^, the formation of which is supported by the Gibbs free energy calculations [46]. During discharge, these peaks gradually shift toward lower binding energies, reflecting a reduction in valance states. I^+^ peak disappears at 1.25 V, accompanied by increased intensity of I_3_⁻ and I_5_⁻. Ultimately, only I⁻ and minor amounts of Cl remain after full discharge. This evolution repeats in subsequent charging, confirming the reversible double-electron transfer of iodine species at the cathode surface (Fig. 5f). Meanwhile, C 1*s* region remains unchanged throughout, indicating only physical adsorption role of porous activated carbon in this period (Fig. S37). On the other hand, *in* situ XRD patterns were performed to investigate phase evolution on cathode. A characteristic peak at 24.4°, corresponding to (111) crystal plane of I_2_, appears at the initial stage of charge. This peak vanishes at the high-voltage range, accompanied by the emergence of a pair of peaks at 31.9° and 48.6°, attributed to the ICl phase. During subsequent discharge, the ICl phase disappears with the re-emergence of the I_2_ phase. Such a reversible evolution was consistently observed over two cycles, confirming the reversibility of iodine redox at the cathode surface (Fig. 5g).

The previously mentioned confinement effect of SP framework on polyiodide ions was validated by resting tests in an H-type cell. As a result, the polyiodides rapidly diffuse through the glass fiber separator within 30 min, whereas SPE setup effectively blocks their migration for at least 48 h (Fig. 5h). Such a difference also pays for distinct Zn plating behaviors on anode surface, as evidenced by SEM images. The anode in SPE exhibits a flat and uniform morphology even after 100 cycles, while fiber residues from the separator and an oxygen distribution from active water molecules are observed in LE, aligning with earlier conclusions (Fig. S38).

Thus, a clear correlation between reversibility and kinetics of cathode surface reaction is established. SPE enhances reversibility by confining polyiodide species and stabilizing interhalogens, which assist in buffering redox fluctuations and further ensuring stable mass transport. In contrast, uncontrollable and complex internal dynamics in LE lead to poor sustainability, where the accumulation of by-products further impairs reaction kinetics.

## Conclusions

Overall, an electrically insulating electrolyte container composed of SiO_2_ and PVDF-*hfp* (SP) was developed to host liquid halogen-ion electrolyte (LE), forming a rigid film (SPE) that enables separator-free Zn-halogen batteries with reversibly customizable electron transfer. Multi-channels supported by microcracks and interparticle gaps provide pathways for ion dissociation, storage, and transport, while strong hydrogen bonding of hydroxyls in SiO_2_ with fluorinated moieties in PVDF-*hfp* disrupts original H_2_O network in LE, thereby modulating the Zn^2+^ solvation as well as buffering concentration gradients and electric fields to promote uniform Zn plating. Meanwhile, SPE demonstrates effective confinement of intermediates, achieving high reversibility and extended lifespan in Zn-halogen batteries across the single-(I^−^/I^0^), double-(I^−^/I^0^/I⁺), and triple-(I^−^/I^0^/I⁺, Cl^−^/Cl^0^) electron transfer mechanisms. Although SPE exhibits slightly weaker mass transfer characteristics than LE, it significantly enhances spatial isolation of polyiodides and interhalogen species for maintaining kinetic stability. Whereas aqueous electrolytes like LE suffer from uncontrollable redox dynamics and by-products accumulation, leading to kinetic deterioration and limited lifespan. This work offers a novel approach for halogen-ion electrolyte design from the perspective of container engineering and provides valuable insights into the interplay between redox reversibility and interfacial reaction kinetics.

## Supplementary Information

Below is the link to the electronic supplementary material.

Supplementary file1 (DOCX 27249 KB)
