# Supplementary material for: Electrically Insulating Rigid Multi-Channel Electrolyte Container for Customizable Electron Transfer in Zn-Halogen Batteries
Source: Nanomicro Lett. 2026 Jan 5;18:168. doi: 10.1007/s40820-025-02007-5 (PMC12765777; doi:10.1007/s40820-025-02007-5)
Supplement: Supplementary file 1 — Supplementary file1 (DOCX 27249 KB) [file 40820_2025_2007_MOESM1_ESM.docx]

Supporting Information for

**Electrically Insulating Rigid Multi-Channel Electrolyte Container for Customizable Electron Transfer in Zn-Halogen Batteries**

Yifan Zhou^1,#^, Yicai Pan^2,#^, Yongqiang Yang^3,^*, Taghreed F. Altamimi^4^, Yunpeng Zhong^5^, Dalal A. Alshammari^6^, Zeinhom M. El-Bahy^7^, Shuquan Liang^1^, Jiang Zhou^1^, and Xinxin Cao^1,^*,

^1^ School of Materials Science and Engineering, Key Laboratory of Electronic Packaging and Advanced Functional Materials of Hunan Province, Central South University, Changsha 410083, P. R. China

^2^ Department of Materials Science and Engineering & Center of Super-Diamond and Advanced Films (COSDAF), City University of Hong Kong, Hong Kong SAR 999077, P. R. China

^3^ Department of Applied Biology and Chemical Technology, The Hong Kong Polytechnic University, Hong Kong SAR 999077, P. R. China

^4^ Department of Physics, College of Science, University of Hail, P.O. Box, 2440, Hail, Saudi Arabia

^5^ Christopher Ingold Laboratory, Department of Chemistry, University College London, London, WC1H0AJ, U.K.

^6^ Department of Chemistry, College of Science, University of Hafr Al Batin, Hafr Al Batin P.O. Box 39524, Saudi Arabia

^7^ Department of Chemistry, Faculty of Science, Al-Azhar University, Nasr City 11884, Cairo, Egypt

^#^ Yifan Zhou and Yicai Pan contributed equally to this work.

* Corresponding authors. E-mail: [yong-qiang.yang@polyu.edu.hk](mailto:yong-qiang.yang@polyu.edu.hk) (Yongqiang Yang); [caoxinxin@csu.edu.cn](mailto:caoxinxin@csu.edu.cn) (Xinxin Cao)

**Supplementary Figures and Tables**


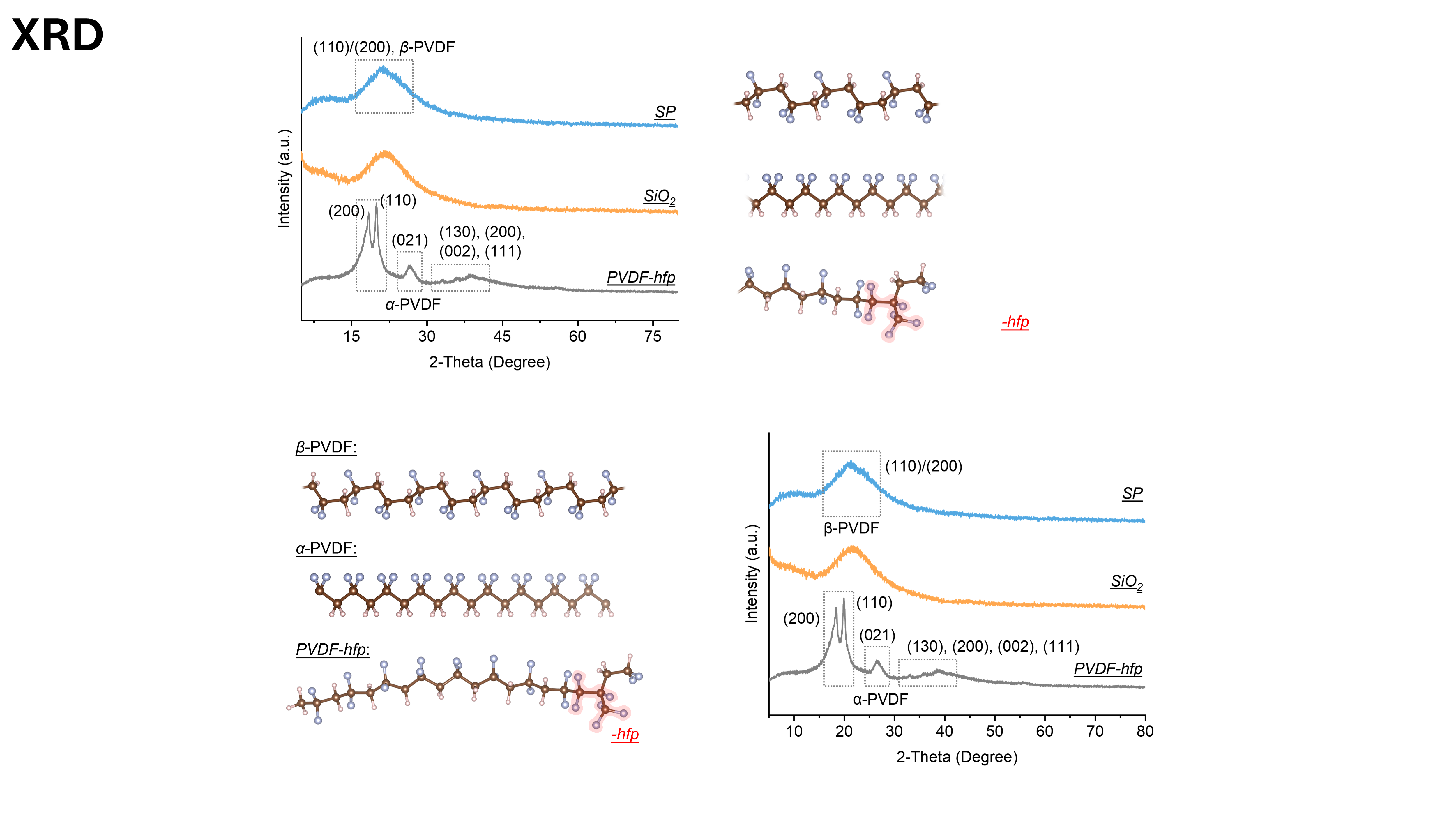


**Fig. S1** Single-chain molecular structure of *α*-PVDF, *β*-PVDF, and PVDF-*hfp*

In the TGTG' conformation of the *α*-phase, the dipole moments along the molecular chains partially cancel each other, resulting in relatively low overall polarization, lower dielectric constant and reduced polarization strength. In contrast, the fully trans TTTT conformation of the *β*-phase allows for effective superposition of dipole moments along the chain direction, leading to higher overall polarization and an increased dielectric constant.

**Fig. S2** XPS spectra of PVDF-*hfp*, SiO_2_ and SP: (**a**) full spectrum, (**b**) O 1*s* and Si 2*p* spectra

**Fig. S3** FT-IR spectra of SiO_2_, PVDF-*hfp*, SP, LE and SPE, respectively

**Fig. S4** Raman spectra of SiO_2_, PVDF-*hfp*, SP, Zn(CF_3_SO_3_)_2_, LE and SPE, respectively


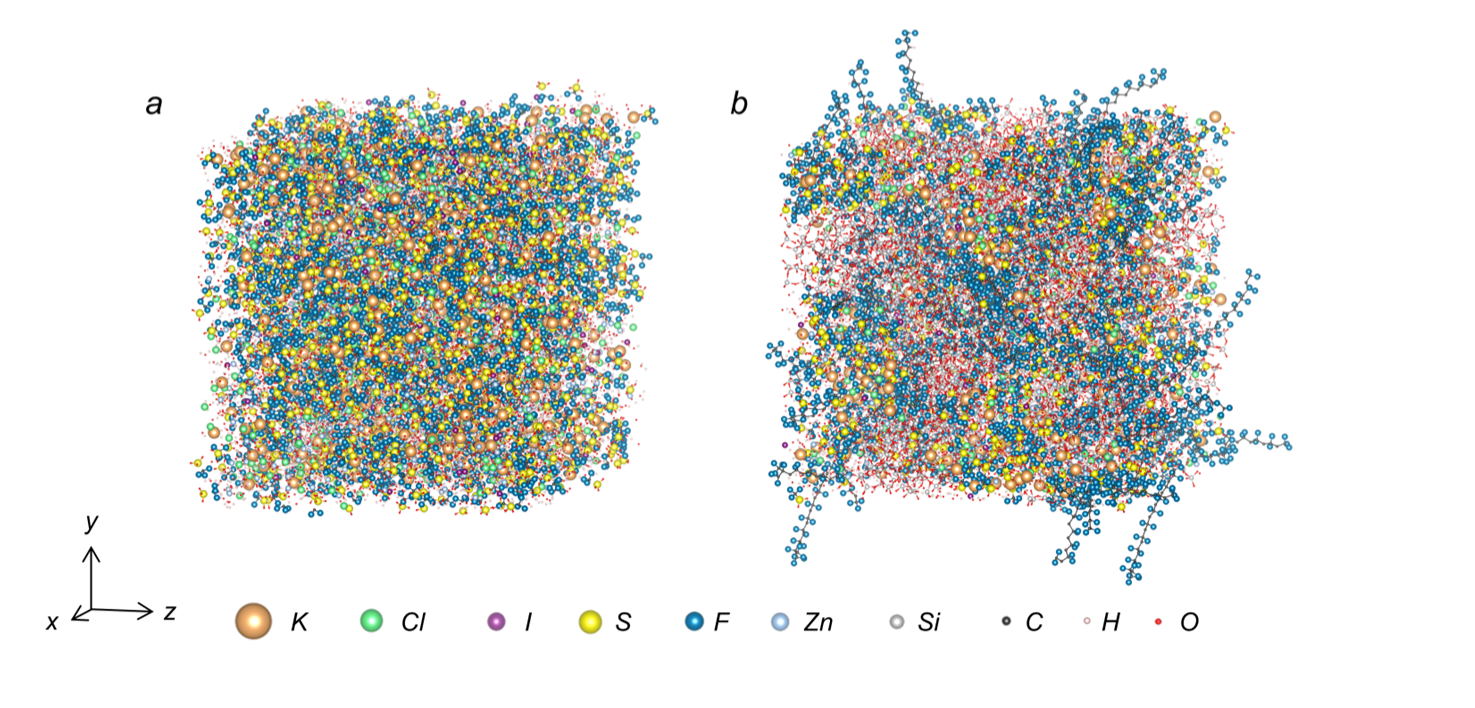


**Fig. S5** 3D snapshots of (**a**) LE and (**b**) SPE obtained from MD simulation

**Fig. S6** TG curves of (**a**) SiO_2_, PVDF-*hfp*, SP powder, SP, SPE, respectively, and (**b**) SPE prepared with different soaking times

TG analysis was conducted under nitrogen at a heating rate of 10°C min^−1^. SiO_2_ exhibits excellent thermal stability, while PVDF-*hfp* begins decomposing at 400°C. The as-prepared SP powder shows an initial mass loss of 32.4% owing to the evaporation of residual NMP, a phenomenon absent in the fully dried SP disc, indicating its effective removal (**Fig. S6a**). To explore the electrolyte absorption kinetics, a more detailed analysis was performed on SPE discs with different soaking times (**Fig. S6b**). The weight loss curves before 450°C can be divided into two distinct stages: the loss of loosely bound “physical water” (below ~300°C) and the loss of more tightly bound “structural water” (300~450 °C). As results, the “physical water” content increases steadily, from 17.5% at 1 hour to 22.1% at 2 hours, before plateauing at 23.0% overnight. These are attributed to a fast adsorption within the macroporous volume, followed by a slower internal diffusion-filling process. On the other hand, the “structural water” content was only around 2% after both 1 and 2 hours but increased significantly to 7.2% after soaking overnight. This indicates that its formation rate is considerably slower. Therefore, to establish stable interactions between the LE components and the SP framework and to allow for the complete formation of ion solvation sheaths, a longer soaking time is evidently required.


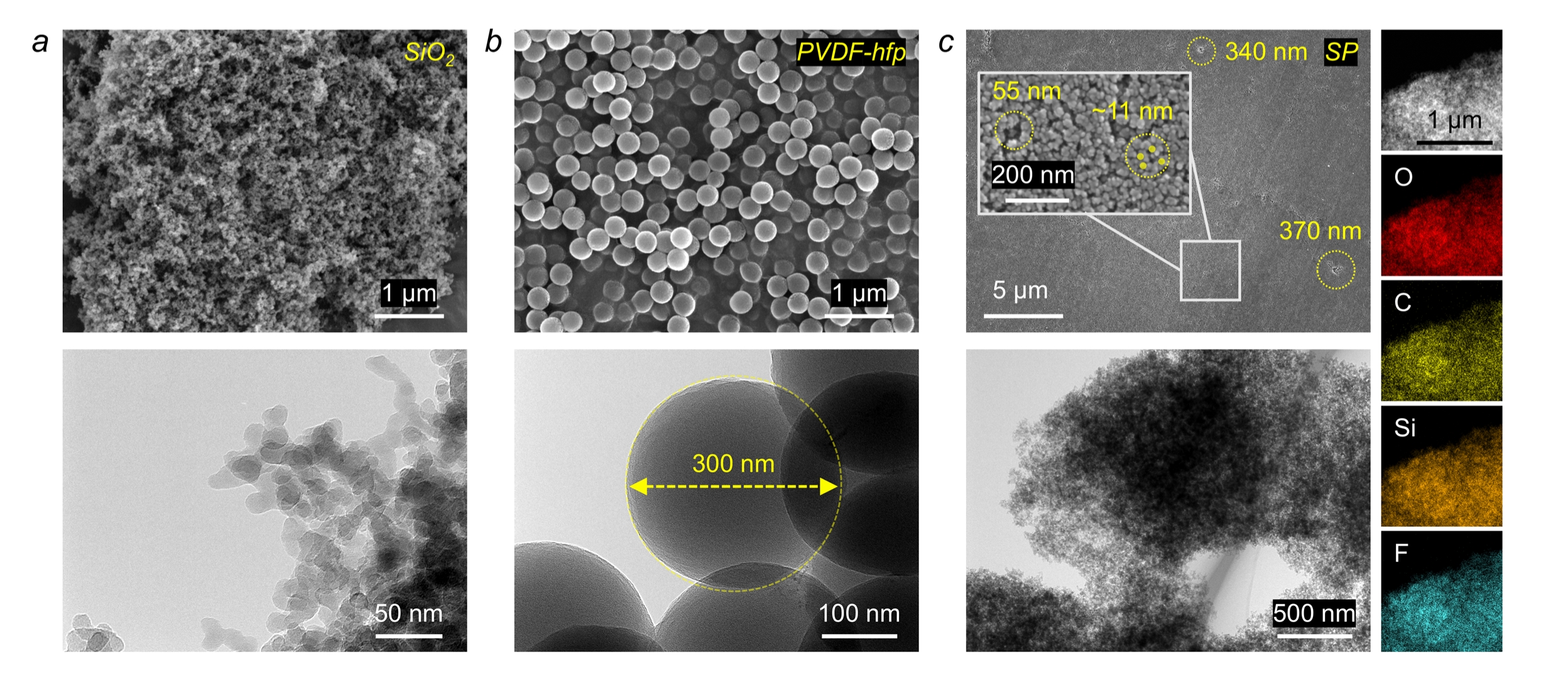


**Fig. S7** SEM and TEM images of (**a**) SiO_2_, (**b**) PVDF-*hfp*; (**c**) SEM and TEM images with EDS elemental mappings of SP surface


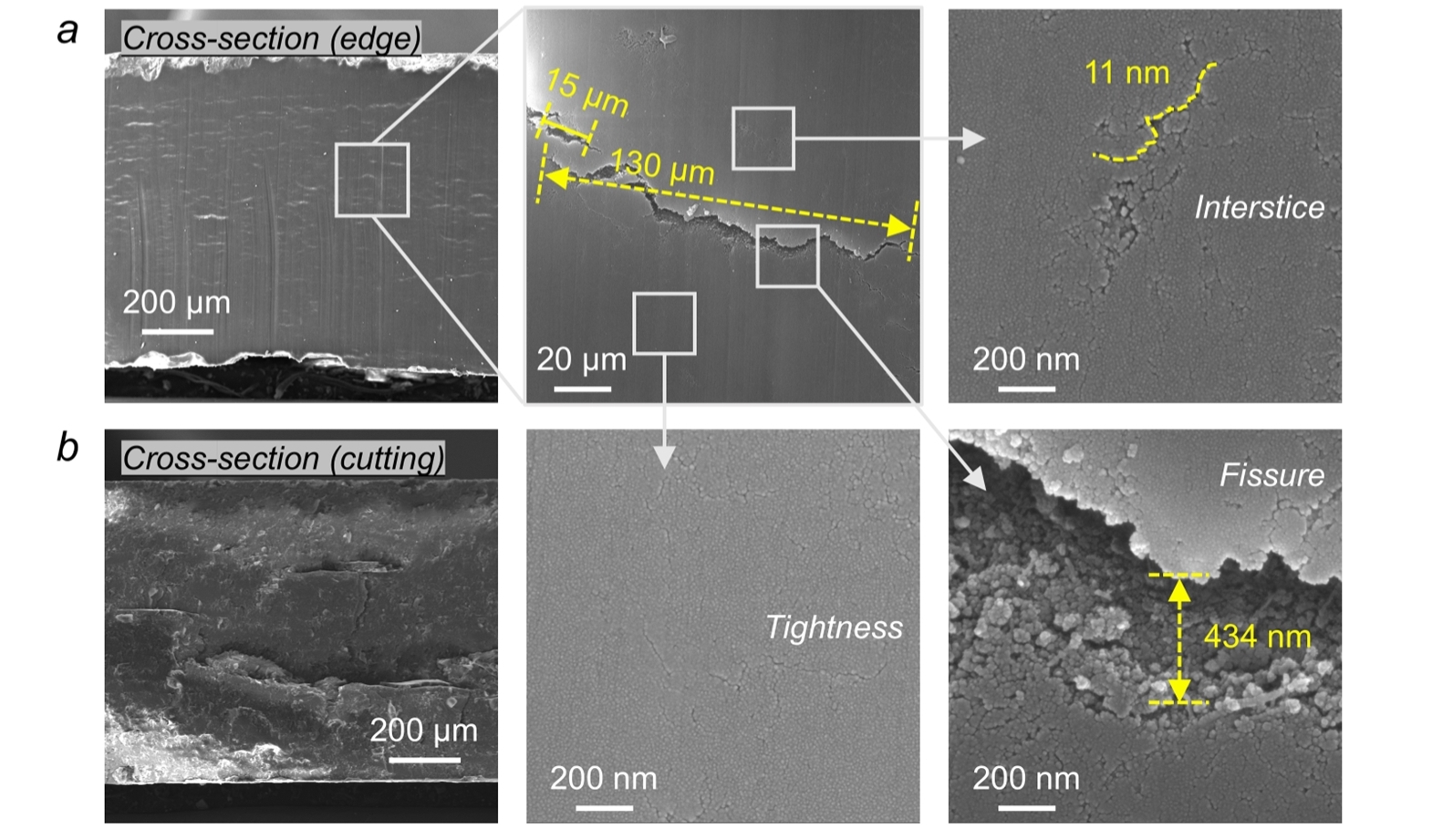


**Fig. S8** Cross-sectional SEM images of SP: (**a**) initial edge, (**b**) cutting edge


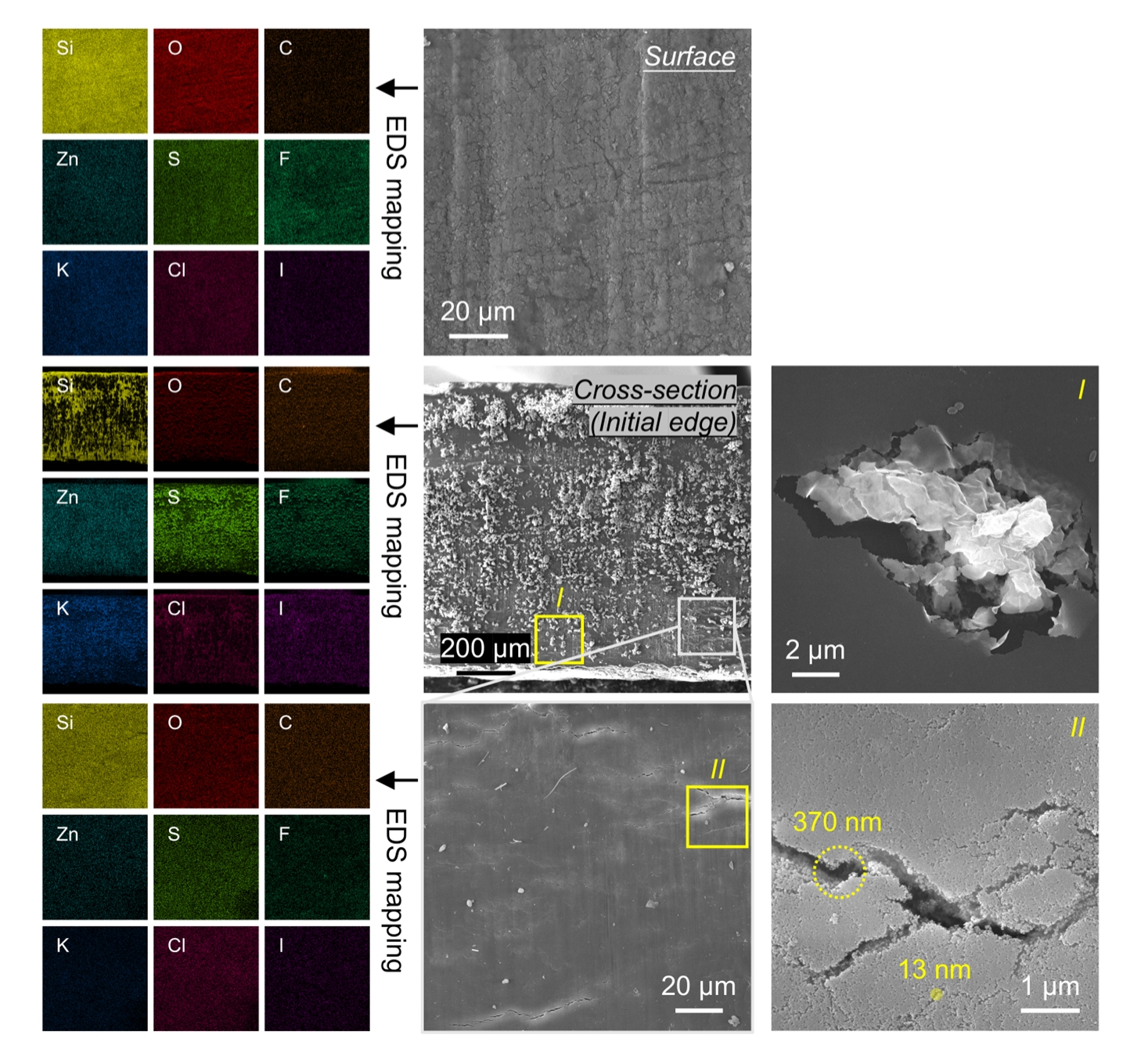


**Fig. S9** Surface and cross-sectional SEM images with EDS elemental mappings of the SPE after heat treatment

**Fig. S10** Nitrogen adsorption-desorption isotherms and corresponding pore size distribution curve of (**a**) SiO_2_, (**b**) PVDF-*hfp*, (**c**) SP, and (**d**) SPE

**Fig. S10a, S10b**: *Type I* adsorption-desorption isotherm.

At the lower relative pressures, the amount adsorbed increases rapidly until saturation is reached, indicating clear *micropore characteristics*.

**Fig. S10c, S10d**: *Type IV* adsorption-desorption isotherm.

As relative pressure increases, the amount adsorbed gradually rises, with a hysteresis phenomenon occurring as the relative pressure approaches 1, which is typical of *mesoporous materials*.

**Fig. S11** Pore size distributions of (**a**) SP and (**b**) SPE obtained from mercury intrusion porosimetry

**Fig. S12** Load-displacement curves of SP and SPE obtained from nanoindentation

Based on the above depth-load curves, the hardness (G Pa) of the samples can be calculated by the following formula:

$$\text{H=}\frac{\text{P}_{\text{max}}}{\text{A}}$$

where *P_max_* is the maximum load (the peak load during the indentation process, *μ*N), and *A* is the contact area (nm^2^) between the indenter tip and sample.

The elastic modulus (G Pa) is determined *via* the Oliver-Pharr method [S1], with the formula:

$$\text{E}_{\text{sample}}\text{=}\left( \frac{\text{1-}\text{ν}_{\text{sample}}^{\text{2}}}{\text{E}_{\text{r}}}\text{-}\frac{\text{1-}\text{ν}_{\text{tip}}^{\text{2}}}{\text{E}_{\text{tip}}} \right)^{\text{-1}}$$

where *ν_sample_* and *ν_tip_* are the Poisson’s ratios of sample and indenter tip, respectively. *E_tip_* is the elastic module of the indenter tip and *E_r_* is the reduced modulus, which was calculated with the formula:

$$\text{E}_{\text{r}}\text{=}\frac{\sqrt{\text{π}}}{\text{2}}\text{⋅}\frac{\text{S}}{\sqrt{\text{A}}}$$

where *S* is the unloading stiffness (*μ*N nm^−1^). The calculation results are summarized in **Table S2**.


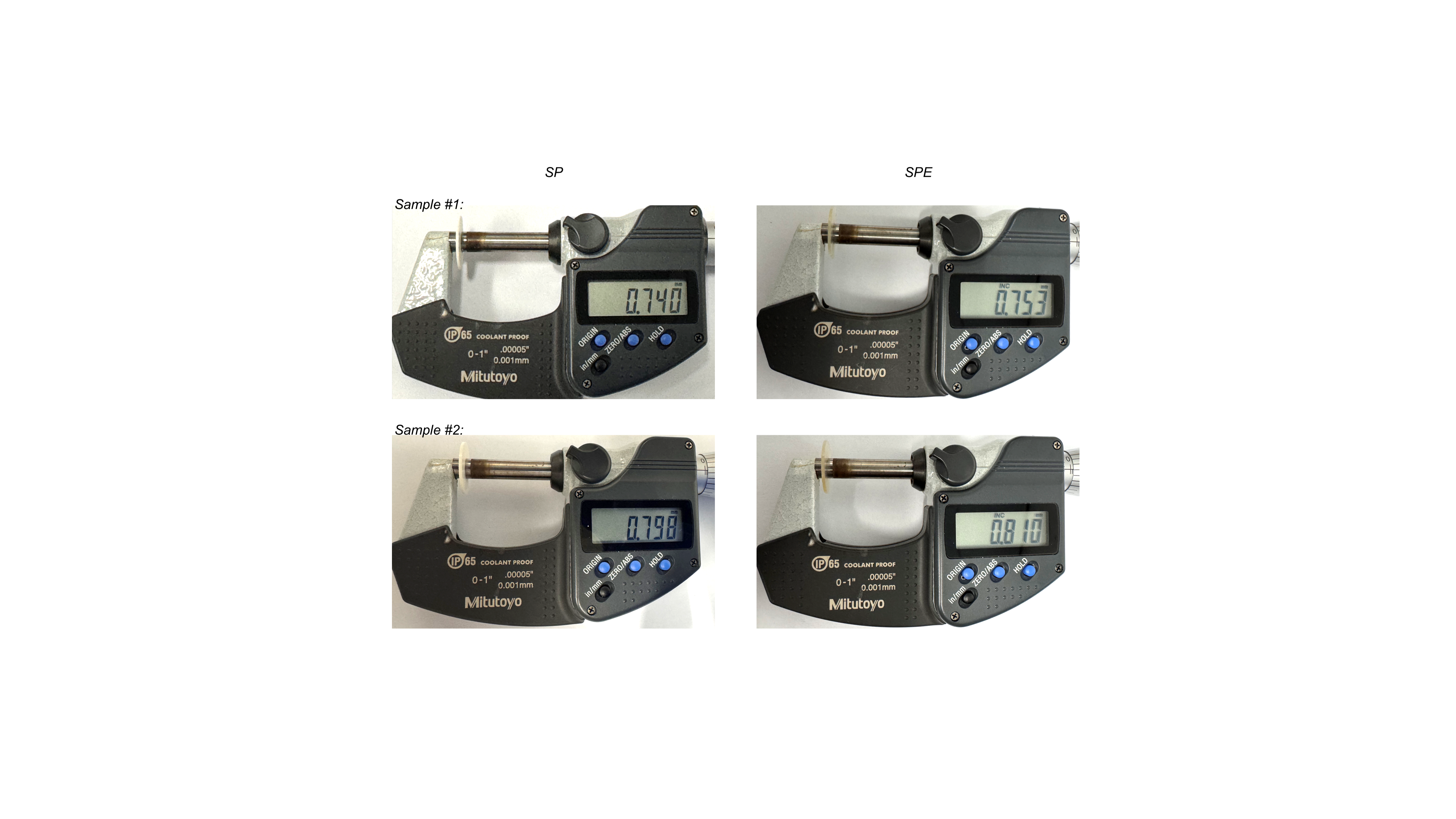


**Fig. S13** Thickness measurements of SP and SPE

**Fig. S14** Frequency-dependent dielectric properties of SiO_2_ and SP

**Fig. S15** EIS plots with stainless steel/stainless steel cells and calculated ionic conductivity of SPE and LE

The ionic conductivity (*σ*) of electrolytes was evaluated *via* EIS using stainless steel/stainless steel cells, which was calculated using the formula:

$$\text{σ=} \frac{\text{l}}{\text{RA}}$$

where *l* is the thickness (cm) of the electrolyte, *A* is the reaction area (cm^2^) between electrolyte and electrodes, *R* is the bulk resistance (Ω) extracted from the high-frequency intercept of the Nyquist plot on the real axis.

**Fig. S16** Chronoamperometry curves with a polarization voltage of 10 mV, corresponding EIS plots before and after the polarization in Zn/Zn cells and calculated Zn^2+^ transference number (*t*) of (**a**) LE and (**b**) SPE

The Zn^2+^ transference number ($\text{t}_{\text{Zn}^{\text{2+}}}$) was determined *via* chronoamperometry (CA) coupled with EIS in Zn/Zn symmetric cells. A polarization voltage ($\text{∆V}$) of 10 mV was applied, and corresponding current relaxation (*I^0^→I^s^*) and interfacial resistance evolution (*R_i_^0^→R_i_^s^*) were monitored. The $\text{t}_{\text{Zn}^{\text{2+}}}$was calculated using the Bruce-Vincent formula [S2]:

$$\text{t}_{\text{Zn}^{\text{2+}}}\text{=}\frac{\text{I}^{\text{s}}\text{ }\left( \text{∆}\text{V - }\text{I}^{\text{0}}\text{R}_{\text{i}}^{\text{0}} \right)}{\text{I}^{\text{0}}\text{ }\left( \text{∆}\text{V - }\text{I}^{\text{s}}\text{R}_{\text{i}}^{\text{s}} \right)}$$

The calculation results are summarized in **Table S3**.

**Fig. S17** *In*-situ differential electrochemical mass spectrometry (DEMS) on the Zn/Zn symmetric cells at 1 mA cm^−2^@0.5 mAh cm^−2^ with (**a**) SPE and (**b**) LE

**Fig. S18** CE plots at 0.5 mA cm^−2^@0.25 mAh cm^−2^ in additional Zn/Cu cells with SPE, with the bottom panels showing the corresponded *avg*. CE with calculated standard deviation

**Fig. S19** Voltage curves for Zn/Cu cells with LE and SPE at Zn plating/stripping conditions of (**a**) 0.5 mA cm^−2^@0.25 mAh cm^−2^, and (**b**) 1 mA cm^−2^@5 mAh cm^−2^

**Fig. S20** Voltage curves at 10 mA cm^−2^@5 mAh cm^−2^ for Zn/Zn cells with SPE and LE


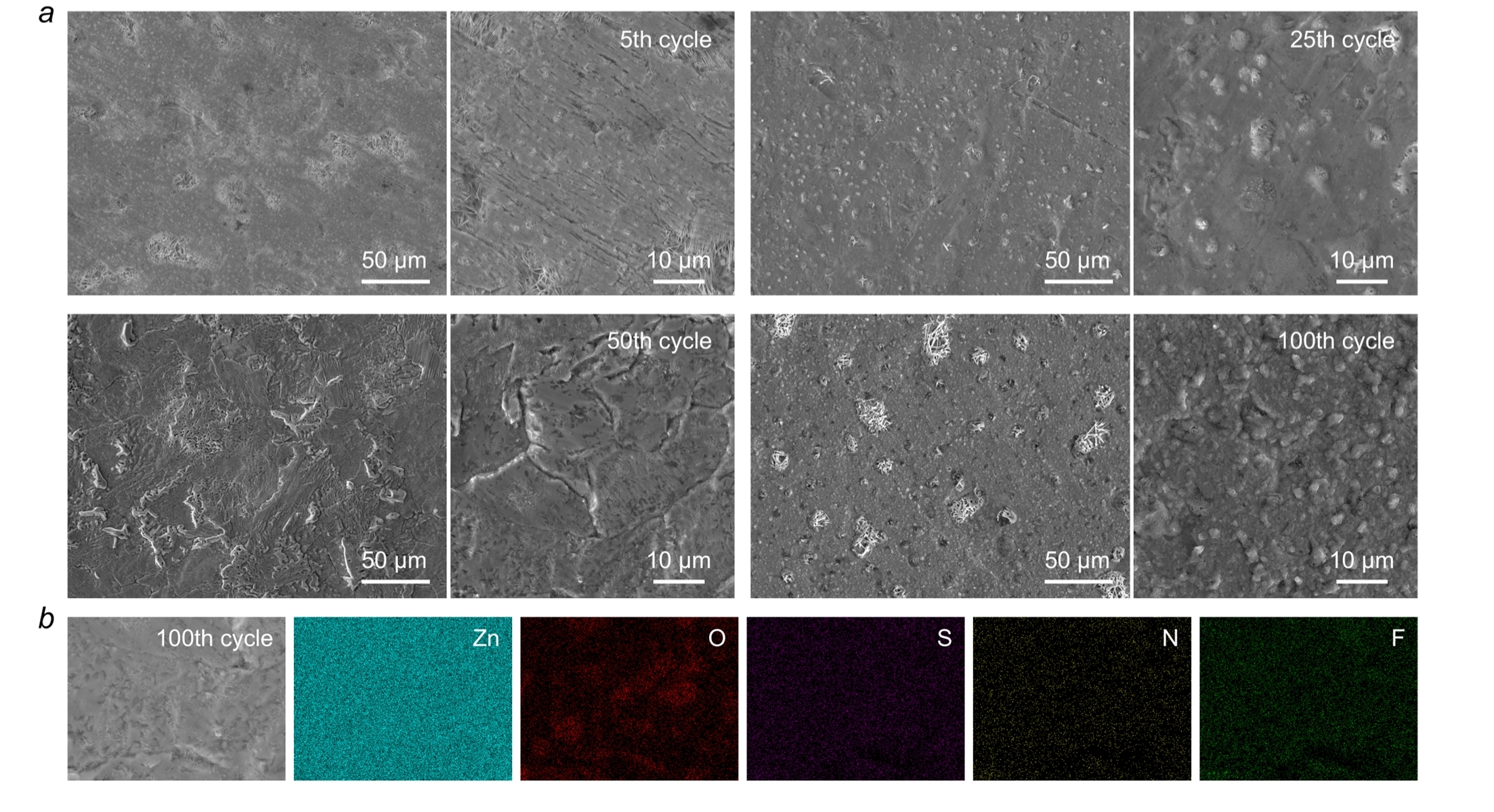


**Fig. S21** (**a**) SEM images after plating for different cycles and (**b**) corresponding EDS mappings in the 100^th^ cycle of Zn electrode in Zn/Zn cells with SPE at 0.5mA cm^−2^@0.25 mAh cm^−2^


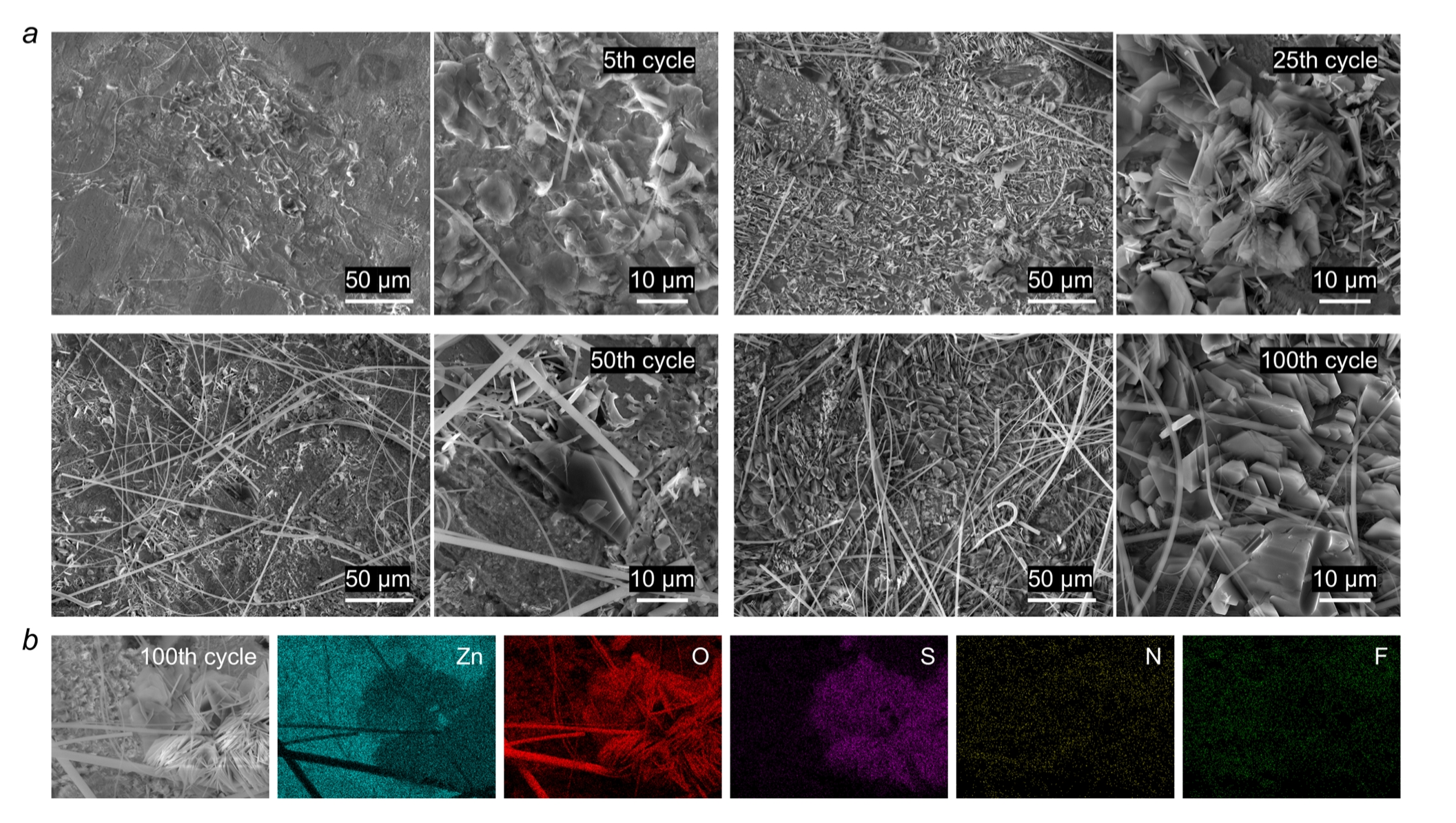


**Fig. S22** (**a**) SEM images after plating for different cycles and (**b**) corresponding EDS mappings in the 100^th^ cycle of Zn electrode in Zn/Zn cells with LE at 0.5mA cm^−2^@0.25 mAh cm^−2^


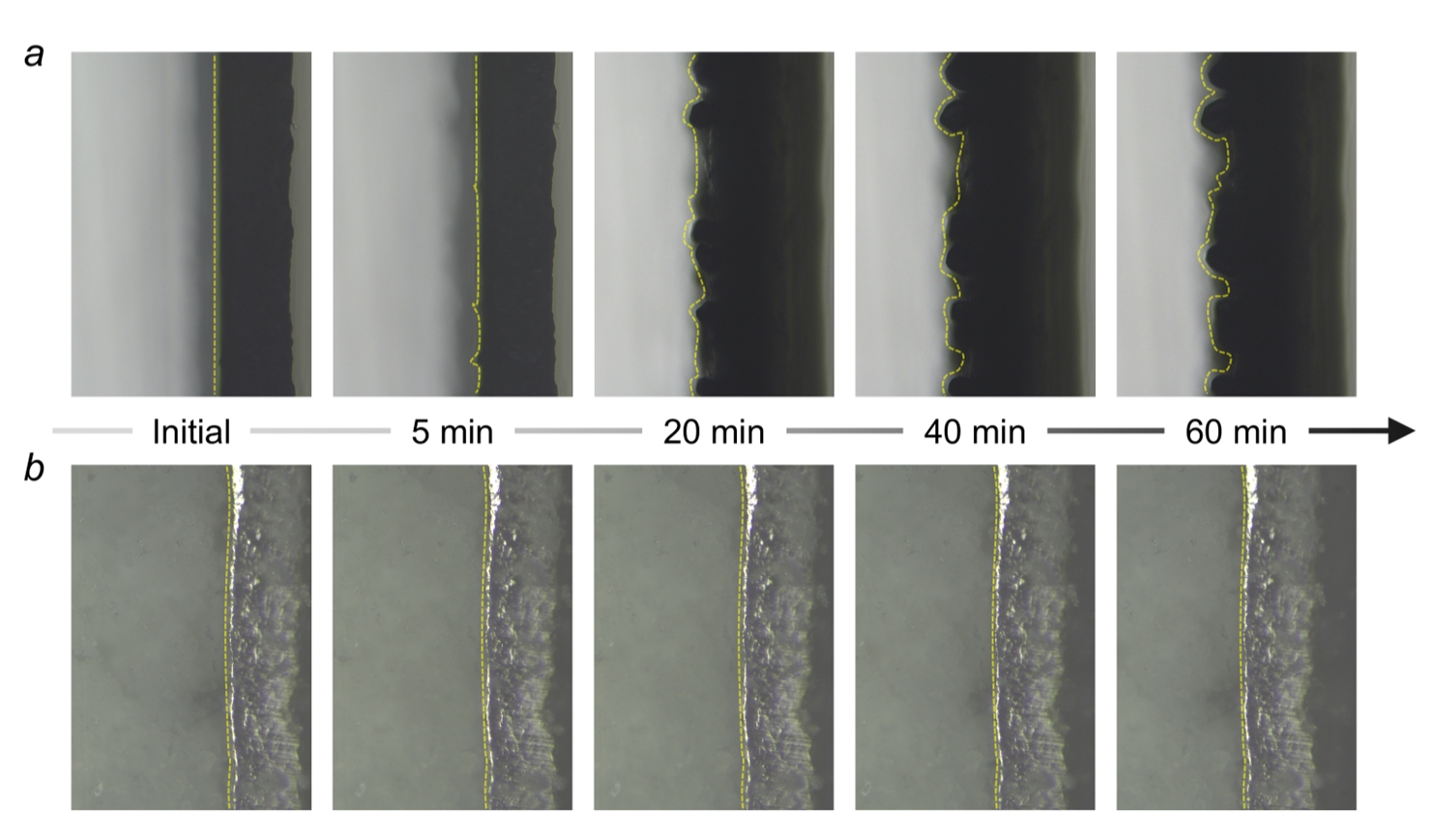


**Fig. S23** *In-situ* optical microscope snapshots of Zn plating in (**a**) LE and (**b**) SPE

**Fig. S24** XRD patterns of Zn electrode after plating for different cycles in Zn/Zn cells with (**a**) SPE and (**b**) LE

**Fig. S25** (**a**) Cycling performance and (**b**) corresponding voltage curve of Zn-halogen coin cell in voltage range of 0.8 to 1.8 V with SPE without potassium chloride and potassium iodide addition

**Fig. S26** (**a**) CV curves at 0.1 to 1.0 mV s^−1^, (**b**) corresponding *log(current) vs. log (scan rate)* plots of redox peaks and (**c**) calculated capacitive-controlled contributions of Zn-halogen cell in the voltage range of 0.8 to 1.9 V with SPE

According to Dunn’s research [S3, S4], the relationship between *current* (*i*) and *scan rate* (*v*) in CV curves can be described by the following equations:

*i = av^b^*

*log(i) = b × log(v) + log(a)*

where *a*, *b* are adjustable parameters, Specifically, a *b*-value of 0.5 indicates a fully diffusion-controlled process, while a *b*-value of 1 represents an entirely capacitive-controlled process. The *b*-value can be directly determined from the slope of the *log(i)* *vs.* *log(v)* plots.

Furthermore, the contributions from pseudocapacitive and diffusion-controlled processes can be quantified by the following equations [S5, S6]:

*i = k_1_v + k_2_v^1/2^*

*i/v^1/2^ = k_1_v^1/2^ + k_2_*

where *k_1_* denotes capacitive contribution, and *k_2_* corresponds to diffusion-controlled contribution. By calculating these parameters (*k_1_* and *k_2_*), relative fractions of capacitive and diffusion-limited currents can be clearly distinguished.

**Fig. S27** Voltage curves for Zn-halogen coin cells in the voltage range of 0.6 to 1.8 V with SPE at (**a**) 3 mA cm^−2^ and (**b**) 10 mA cm^−2^

**Fig. S28** (**a**) Cycling performance of Zn-halogen coin cell in the voltage range of 0.6 to 1.8 V with SPE at 20 mA cm^−2^; (**b**) Cycling performances of additional coin cells, with the bottom panels showing the corresponded *avg*. CE with calculated standard deviation

**Fig. S29** Voltage curves for Zn-halogen pouch cells (3×3 cm^2^) in the voltage range of 0.8 to 1.8 V: (**a**) SPE and (**b**) LE with carbon fiber cloth current collector at 4 mA cm^−2^; (**c**) SPE with carbon felt current collector at 2 mA cm^−2^

**Fig. S30** *In*-situ DEMS on the Zn-halogen coin cell in the voltage range of 0.8 to 2.1 V at 3 mA cm^−2^ with (**a**) SPE and (**b**) LE


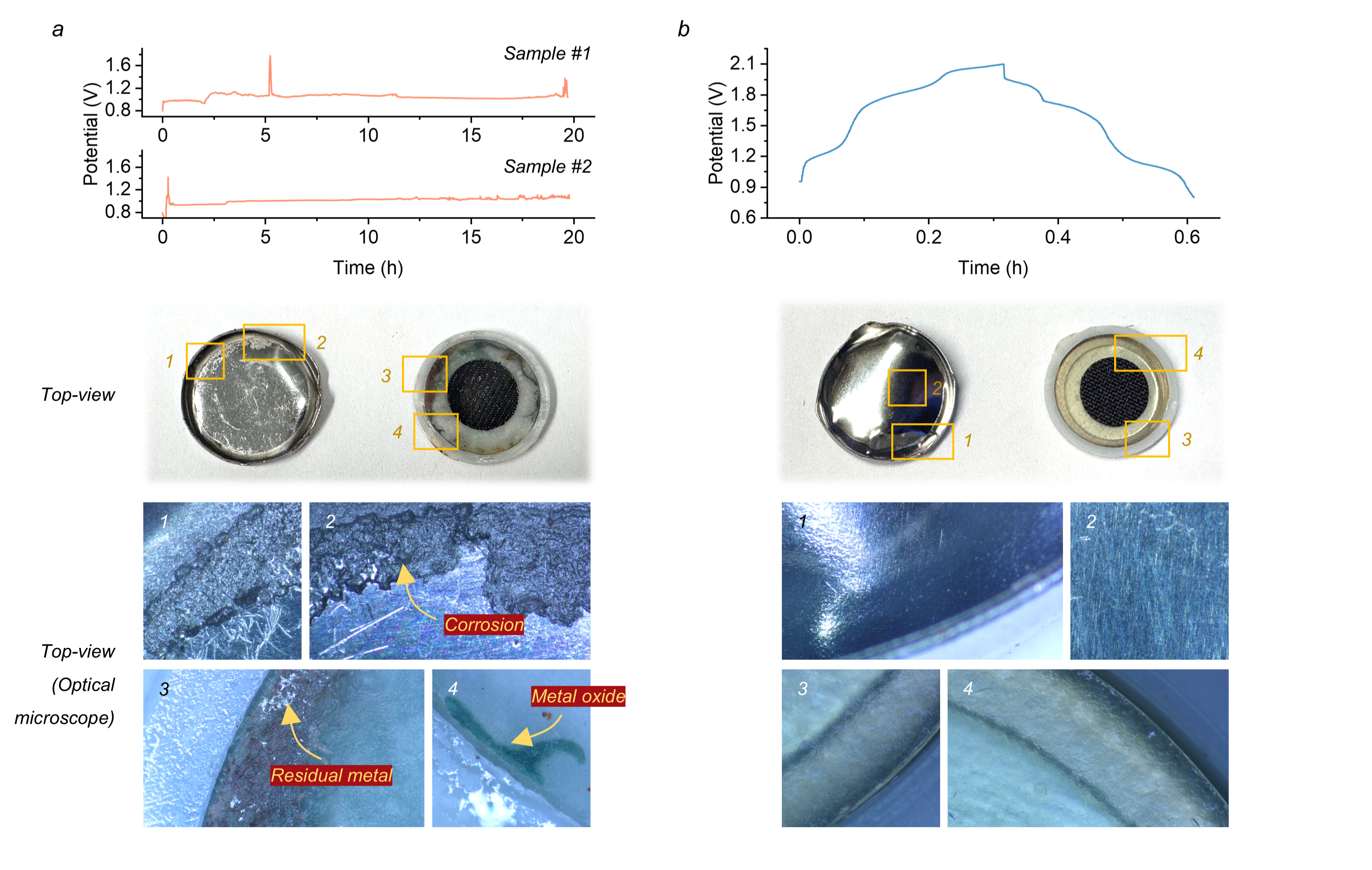


**Fig. S31** Voltage curves and optical images of the battery components after cycling of Zn-halogen coin cells in the voltage range of 0.8 to 2.1 V with (**a**) LE and (**b**) SPE at 1 mA cm^−2^

**Fig. S32** Cycling performances of additional Zn-halogen coin cells in the voltage range of 0.8 to 2.1 V with SPE at 10 mA cm^−2^, with the bottom panels showing the corresponded *avg*. CE with calculated standard deviation

**Fig. S33** Voltage curves of Zn-halogen coin cells in the voltage range of 0.8 to 2.1 V with SPE at (**a**) 3 mA cm^−2^ and (**b**) 10 mA cm^−2^

**Fig. S34** Comparisons of areal capacity, coulombic efficiency and lifespan in other metal-halogen coin cells

**Fig. S35** Galvanostatic intermittent titration technique (GITT) voltage curves of Zn-halogen coin cells in the voltage range of 0.8 to 1.8 V with SPE and LE at 3 mA cm^−2^

The GITT was performed in coin cells with a current pulse duration of 300 s and a relaxation time of 600 s. The diffusion coefficient (*D*) can be calculated according to the following equation [S7]:

$$\text{D}\text{ =} \frac{\text{4}\text{L}^{\text{2}}}{\text{πτ}}\left( \frac{\text{∆}\text{E}_{\text{s}}}{\text{∆}\text{E}_{\text{t}}} \right)^{\text{2}}$$

where *t* and 𝜏 represent the durations of current pulse (s) and relaxation period (s), respectively; *L* is the diffusion length of charge carrier, equal to the electrode thickness; *∆E_s_* is the steady-state voltage change (V) induced by current pulse, and *∆E_t_* is the voltage variation (V) during the constant current pulse after subtracting the voltage change following relaxation.

**Fig. S36** *Ex*-situ EIS spectra of double-electron transfer Zn-halogen coin cells with SPE and LE

**Fig. S37** *Ex-situ* XPS spectra of cathodes in the different cycling states in double-electron transfer Zn-halogen cells with SPE: (**a**) Full spectra; (**b**) C 1*s* region


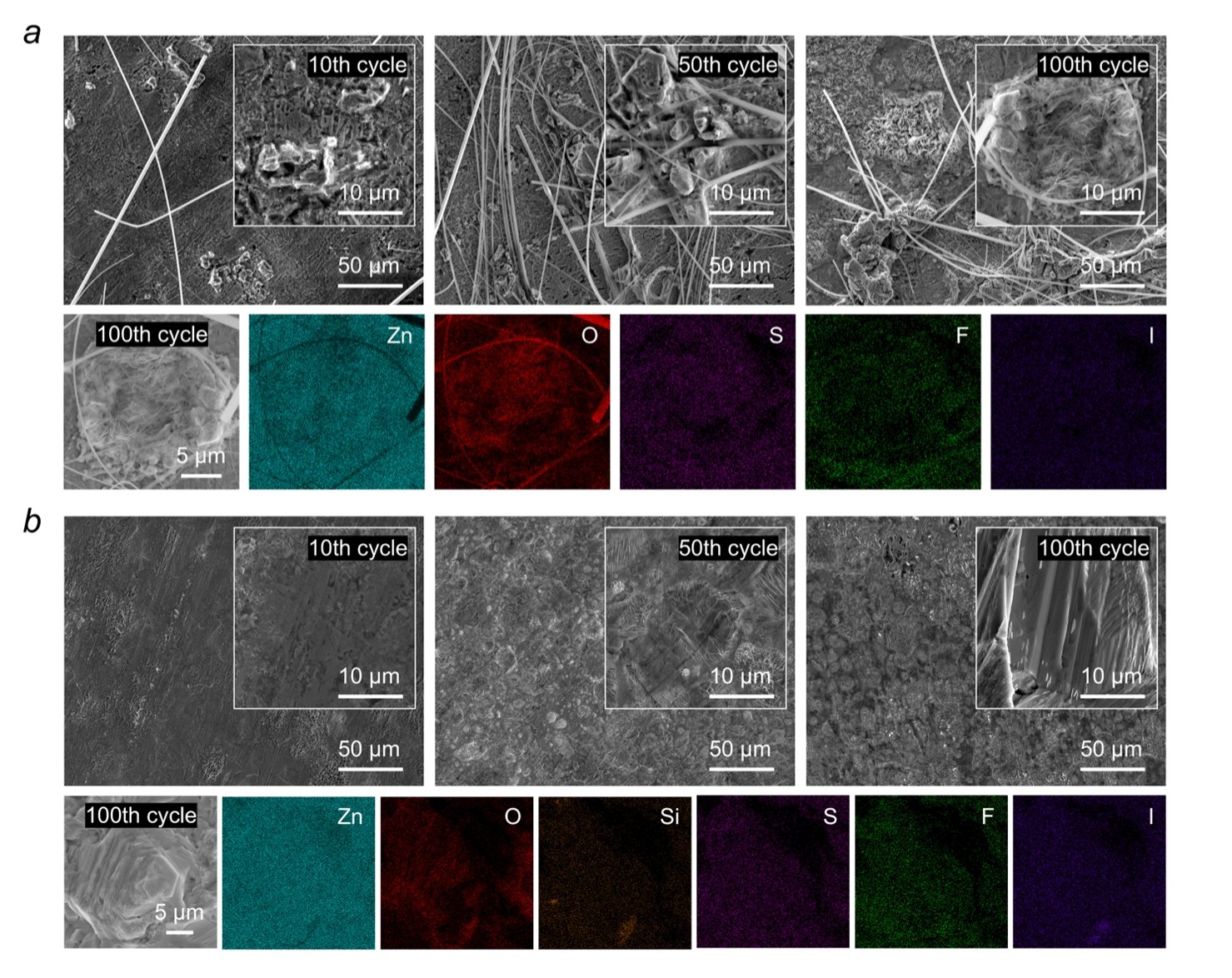


**Fig. S38** SEM images for different cycles and corresponding EDS mappings in the 100^th^ cycle of Zn anode in double-electron transfer Zn-halogen coin cells with (**a**) LE and (**b**) SPE, respectively

**Table S1** Hydrogen bond statistics derived from the solvation boxes of SP and SPE

| ***Sample*** | ***Types of H-bonds*** | ***Lengths* (Å)** | | | | ***Numbers*** |
| --- | --- | --- | --- | --- | --- | --- |
|  |  | *Min.* | *Max.* | *Md.* | *Avg.* |  |
| LE | H_2_O−CF_3_SO_3_^−^ | 1.37505 | 2.49994 | 2.13763 | 2.09969 | 5509 (54%) |
|  | H_2_O−H_2_O | 1.35916 | 2.49983 | 1.98025 | 1.99027 | 4747 (46%) |
| SPE | H_2_O−CF_3_SO_3_^−^ | 1.34828 | 2.49916 | 1.80388 | 1.87974 | 3291 (36%) |
|  | H_2_O−H_2_O | 1.43786 | 2.49916 | 1.85446 | 1.90428 | 1651 (18%) |
|  | H_2_O−SiO_2_ | 1.43786 | 2.49916 | 2.01224 | 2.02149 | 2548 (27%) |
|  | H_2_O−PVDF-*hfp* | 1.43786 | 2.49916 | 1.87967 | 1.92704 | 1764 (19%) |

**Table S2** Hardness and elastic modulus of SP and SPE derived from nanoindentation

| ***Sample*** | ***No.*** | ***P_max_ (μN)*** | ***A (nm^2^)*** | ***S (μN mm^−1^)*** | ***E (GPa)*** | ***H (GPa)*** |
| --- | --- | --- | --- | --- | --- | --- |
| SP | 1 | 1996.75 | 8576600.71 | 10.70 | 3.238109 | 0.232814 |
|  | 2 | 1996.87 | 6814426.37 | 9.77 | 3.315582 | 0.293036 |
|  | 3 | 1996.89 | 8120832.23 | 11.20 | 3.481248 | 0.245898 |
|  | 4 | 1996.71 | 8995816.70 | 10.32 | 3.047446 | 0.221960 |
|  | 5 | 1996.99 | 7832634.02 | 11.04 | 3.495007 | 0.254957 |
| SPE | 1 | 1996.76 | 7905203.69 | 9.84 | 3.101648 | 0.252588 |
|  | 2 | 1996.87 | 6934619.92 | 8.57 | 2.881734 | 0.287957 |
|  | 3 | 1996.83 | 9188505.38 | 9.45 | 2.762539 | 0.217318 |
|  | 4 | 1996.89 | 9225901.08 | 9.44 | 2.753576 | 0.216444 |
|  | 5 | 1996.94 | 9821988.13 | 10.29 | 2.910396 | 0.203313 |

**Table S3**. Calculations of Zn^2+^ transference numbers (*t*) for LE and SPE

| ***Sample*** | ***I^0^* (mA)** | ***I^s^* (mA)** | ***R_i_^0^* (Ω)** | ***R_i_^s^* (Ω)** | ***t(Zn^2+^)*** |
| --- | --- | --- | --- | --- | --- |
| LE | 0.432 | 0.068 | 88.65 | 126.50 | 0.70142 |
| SPE | 0.440 | 0.099 | 73.86 | 127.60 | 0.57912 |

**Table S4** Comparisons of areal capacity, discharge mid-voltage and capacity in other metal-halogen pouch cells

| ***Label*** | ***Type*** | ***Electrochemical performance*** | | | | ***Refs.*** |
| --- | --- | --- | --- | --- | --- | --- |
|  |  | *Capacity (mAh)* | *Areal capacity (mAh cm^−2^)* | *Current density (mA cm^−2^)* | *Mid-voltage (V)* |  |
| AC//3 M Zn(CF_3_SO_3_)_2_ + 2 M KCl+0.5 M KI in SPE | I^−^/I^0^/I^+^ | 107 | 11.9 | 2 | 1.3 | *This work* |
| I_2_@EI-ZrP//HCP | I^−^/I^0^ | 10 | 1.11 |  | 1.2 | [S8] |
| PC@Fe_2_N-I_2_//2 M ZnSO_4_ | I^−^/I^0^ | 14.2 | 0.75 | 1.85 | 1.07 | [S9] |
| I_2_@CNT//PAH-PCH/CP | I^−^/I^0^ | 20 | 2.22 |  | 1.15 | [S10] |
| I_2_@Fe SAC-MNC//2 M ZnSO_4_ + 0.04 M I_3_^−^ | I^−^/I^0^ | 36.88 |  |  | 1.21 | [S11] |
| CNT@MPC12-I^−^//1 M ZnSO_4_ | I^−^/I^0^ | 51.3 | 1.71 | 5 | 1.18 | [S12] |
| I_2_@Ti-C//2 M ZnSO_4_ +  5 mM TDFND | I^−^/I^0^ | 68 | 2.6 | 4 | 1.08 | [S13] |
| I_2_@PC//2 M ZnSO_4_ + PG | I^−^/I^0^ | 78.5 |  |  | 1.15 | [S14] |
| I_2_@AC//2 M ZnSO_4_//Zn-CCS | I^−^/I^0^ | 140 | 4.39 | 4 | 0.98 | [S15] |
| NH_4_V_4_O_10_+PAC//3 M Zn(CF_3_SO_3_)_2_ + 0.5 M KI in EG/H_2_O | I^−^/I^0^ | 2.17 | 0.241 | 1.5 | 1.08 | [S16] |
| I_2_@AC//0.5 M ZnSO_4_ + 0.5 M Li_2_SO_4_ | I^−^/I^0^ | 12.9 | 0.516 | 0.806 | 1.15 | [S17] |
| CC-I_2_//3 m ZnSO_4_ + 3.5 m LiBr + 0.1 m LiNO_3_ | I^−^/I^0^/I^+^ | 0.799 | 2.22 | 1.5 | 1.4 | [S18] |
| I_2_@C//ZTEs | I^−^/I^0^/I^+^ | 10 | 0.25 |  | 1.3 | [S19] |
| PTCDI//I_2_ + saturated KCl | I^−^/I^0^/I^+^ | 2.72 | 0.057 | 80 | 1.25 | [S20] |
| I_2_@PAC//19 m ZnCl_2_ +  19 m LiCl + 8 m ACN | I^−^/I^0^/I^+^ | 2.436 | 3.1 | 0.8 | 1.4 | [S21] |
| I_2_@PAC//2 M ZnCl_2_ + PEG | I^−^/I^0^/I^+^ | 67.2 | 4.2 | 4 | 1.3 | [S22] |
| I_2_@AC//BAVBr | I^−^/I^0^/I^+^ | 19.8 | 1.65 | 3 | 1.25 | [S23] |
| IBr@CF//2 M ZnSO_4_ | I^−^/I^0^/I^+^, Br^−^/Br^0^ | 110 | 3.67 | 8.33 | 1.5 | [S24] |
| I_2_@AC//30 m ZnCl_2_ | I^−^/I^0^/I^+^, Cl^−^/Cl^0^ | 124.7 | 1.56 | 2.5 | 1.48 | [S25] |

**Table S5** Comparisons in mass transfer capacities and interfacial reaction stability with PVDF-*hfp* gels, Janus separators, quasi-solid electrolytes used interphase-engineering approaches for Zn-based batteries

***i) PVDF-hfp gels***

| ***Material*** | ***σ (mS cm^−1^)*** | ***t (Zn^2+^)*** | ***ESW (V)*** |
| --- | --- | --- | --- |
| Zn(TFSI)_2_+0.1EC+ 0.9PEGDME+PVDF-*hfp* | 0.47 | − | >3 |
| Zn(Tf)_2_+ PVDF-*hfp* solid polymer | 0.0244 | 0.983 | 3.45 |

*continuation of the previous table*

| ***CE (%)***  ***in half-cell*** | ***Lifespan (h)***  ***in half-cell*** | ***Lifespan (h)***  ***in Zn-Zn cell*** | ***Full cell*** | ***Lifespan*** | ***Refs.*** |
| --- | --- | --- | --- | --- | --- |
| − | − | − | − | − | [S26] |
| − | − | 100 (0.05@0.05) | − | − | [S27] |

***ii) Janus separators***

| ***Material*** | ***σ (mS cm^−1^)*** | ***t (Zn^2+^)*** | ***ESW (V)*** |
| --- | --- | --- | --- |
| 3D VG on glass fiber separator | − | − | − |
| Graphene and sulfonic cellulose on glass fiber | 2.45×10^−5^ | 0.826 | − |
| Bacterial cellulose (BC)//AgNWs-BC | 3.83 | − | − |
| Dowex//Fe-SCNT-GF | 14.6 | 0.629 | − |
| PAZPM hydrogel | 17.0 | − | − |

*continuation of the previous table*

| ***CE (%)***  ***in half-cell*** | ***Lifespan (h)***  ***in half-cell*** | ***Lifespan (h)***  ***in Zn-Zn cell*** | ***Full cell*** | ***Lifespan*** | ***Ref.*** |
| --- | --- | --- | --- | --- | --- |
| − | − | 600 (10@1) | Zn−V_2_O_5_ | 5000 (5 A g^−1^) | [S28] |
| 99.6 | 1780 (1@1) | 1400 (10@10) | Zn−CNT-MnO_2_ | 1900 (1) | [S29] |
| 98.1 | >800 (1@1) | 600 (10@1) | Zn−K_0.27_Mn_2_·0.54H_2_O | 700 (3) | [S30] |
| 99.5 | 1000 (2@1) | 2500 (1@1) | Zn−I_2_-AC | 30000 (5) | [S31] |
| − | − | 3600 (0.5@0.25) | Zn−(NH_4_)_2_V_10_O_25_·8H_2_O | 1000 (5) | [S32] |

***iii) Quasi-solid electrolytes used interphase-engineering approaches***

| ***Material*** | ***σ (mS cm^−1^)*** | ***t (Zn^2+^)*** | ***ESW (V)*** |
| --- | --- | --- | --- |
| ZnSO_4_-PEG400 hydrogel | − | − | − |
| ZnAc_2_-PVA416 hydrogel | 49.8 | 0.517 | − |
| ZnAc_2_-PVA hydrogel | 16.5 | − | − |
| Zn(OTf)_2_ in cellulose gel | 38.6 | 0.73 | − |
| ZnSO_4_ in CS- and PASP-gels | 5.51 | − | − |
| CarraChi gel | 5.3 | 0.52 | − |
| CD-PEO/PAM hydrogel | 22.4 | 0.923 | − |
| PAM-Hbimcp-Zn hydrogel | 38.2 | − | − |
| PAAm/DMSO/Zn(Otf)_2_ hydrogel | − | − | − |

*continuation of the previous table*

| ***CE (%)***  ***in half-cell*** | ***Lifespan (h)***  ***in half-cell*** | ***Lifespan (h)***  ***in Zn-Zn cell*** | ***Full cell*** | ***Lifespan*** | ***Refs.*** |
| --- | --- | --- | --- | --- | --- |
| 99.96 | 200 (12@1) | >1500 (18@18) | Zn−MnO_2_ | 200 (0.07 A g^−1^) | [S33] |
| − | − | >4 (0.5@0.1) | Zn−PANI | 5000 (2) | [S34] |
| − | − | 1450 (0.5@0.5) | Zn−ZnHCF | 700 (0.2 A cm^−2^) | [S35] |
| 99.4 | 400 (5@1) | 2000 (0.5@0.5) | Zn−PANC/CC | 2000 (2) | [S36] |
| 99.6 | >800 (10@5) | 2200 (10@10) | Zn−MnO_2_ | 5000 (5) | [S37] |
| 99.5 | 120 (5@1) | 4000 (10@35) | Zn−Zn*_x_*V_2_O_5_ (0.9 Ah) | 200 (0.2) | [S38] |
| − | − | 180 (1@1) | Zn−La-V_2_O_5_ | 3500 (5) | [S39] |
| − | − | 500 (1@1) | Zn−V_2_O_5_ | 1000 (2) | [S40] |
| 99.5 | 1300 (2@4) | 1350 (2@4) | Zn−Zn_3_V_2_O_8_ | 3000 (1) | [S41] |

**Supplementary References**

1. Measurement of thin film mechanical properties using nanoindentation. MRS Bull. **17**(7), 28–33 (1992). <https://doi.org/10.1557/S0883769400041634>
2. P.G. Bruce, J. Evans, C.A. Vincent, Conductivity and transference number measurements on polymer electrolytes. Solid State Ion. **28**, 918–922 (1988). <https://doi.org/10.1016/0167-2738(88)90304-9>
3. V. Augustyn, J. Come, M.A. Lowe, J.W. Kim, P.-L. Taberna et al., High-rate electrochemical energy storage through Li+ intercalation pseudocapacitance. Nat. Mater. **12**(6), 518–522 (2013). <https://doi.org/10.1038/nmat3601>
4. V. Augustyn, P. Simon, B. Dunn, Pseudocapacitive oxide materials for high-rate electrochemical energy storage. Energy Environ. Sci. **7**(5), 1597 (2014). <https://doi.org/10.1039/c3ee44164d>
5. T. Brezesinski, J. Wang, S.H. Tolbert, B. Dunn, Ordered mesoporous alpha-MoO_3_ with *Iso*-oriented nanocrystalline walls for thin-film pseudocapacitors. Nat. Mater. **9**(2), 146–151 (2010). <https://doi.org/10.1038/nmat2612>
6. D. Chao, C. Zhu, P. Yang, X. Xia, J. Liu et al., Array of nanosheets render ultrafast and high-capacity Na-ion storage by tunable pseudocapacitance. Nat. Commun. **7**, 12122 (2016). <https://doi.org/10.1038/ncomms12122>
7. W. Weppner, R.A. Huggins, Determination of the kinetic parameters of mixed-conducting electrodes and application to the system Li_3_Sb. J. Electrochem. Soc. **124**(10), 1569–1578 (1977). <https://doi.org/10.1149/1.2133112>
8. J. Wu, J.-L. Yang, B. Zhang, H.J. Fan, Immobilizing polyiodides with expanded Zn^2+^ channels for high-rate practical zinc-iodine battery. Adv. Energy Mater. **14**(3), 2302738 (2024). <https://doi.org/10.1002/aenm.202302738>
9. Q. Chen, S. Chen, J. Ma, S. Ding, J. Zhang, Synergic anchoring of Fe2N nanoclusters on porous carbon to enhance reversible conversion of iodine for high-temperature zinc-iodine battery. Nano Energy **117**, 108897 (2023). <https://doi.org/10.1016/j.nanoen.2023.108897>
10. J.-L. Yang, Z. Yu, J. Wu, J. Li, L. Chen et al., Hetero-polyionic hydrogels enable dendrites-free aqueous Zn-I(2) batteries with fast kinetics. Adv. Mater. **35**(44), e2306531 (2023). <https://doi.org/10.1002/adma.202306531>
11. X. Yang, H. Fan, F. Hu, S. Chen, K. Yan et al., Aqueous zinc batteries with ultra-fast redox kinetics and high iodine utilization enabled by iron single atom catalysts. Nanomicro Lett. **15**(1), 126 (2023). <https://doi.org/10.1007/s40820-023-01093-7>
12. J. He, H. Hong, S. Hu, X. Zhao, G. Qu et al., Chemisorption effect enables high-loading zinc-iodine batteries. Nano Energy **119**, 109096 (2024). <https://doi.org/10.1016/j.nanoen.2023.109096>
13. T. Li, S. Hu, C. Wang, D. Wang, M. Xu et al., Engineering fluorine-rich double protective layer on Zn anode for highly reversible aqueous zinc-ion batteries. Angew. Chem. Int. Ed. **62**(51), e202314883 (2023). <https://doi.org/10.1002/anie.202314883>
14. J. Hao, L. Yuan, Y. Zhu, X. Bai, C. Ye et al., Low-cost and non-flammable eutectic electrolytes for advanced Zn-I(2) batteries. Angew. Chem. Int. Ed. **62**(39), e202310284 (2023). <https://doi.org/10.1002/anie.202310284>
15. Z. Hu, X. Wang, W. Du, Z. Zhang, Y. Tang et al., Crowding effect-induced zinc-enriched/water-lean polymer interfacial layer toward practical Zn-iodine batteries. ACS Nano **17**(22), 23207–23219 (2023). <https://doi.org/10.1021/acsnano.3c10081>
16. Y. Yang, S. Guo, Y. Pan, B. Lu, S. Liang et al., Dual mechanism of ion (de)intercalation and iodine redox towards advanced zinc batteries. Energy Environ. Sci. **16**(5), 2358–2367 (2023). <https://doi.org/10.1039/D3EE00501A>
17. K. Wang, H. Li, Z. Xu, Y. Liu, M. Ge et al., An iodine-chemisorption binder for high-loading and shuttle-free Zn–iodine batteries. Adv. Energy Mater. **14**(17), 2304110 (2024). <https://doi.org/10.1002/aenm.202304110>
18. S. Lv, T. Fang, Z. Ding, Y. Wang, H. Jiang et al., A high-performance quasi-solid-state aqueous zinc-dual halogen battery. ACS Nano **16**(12), 20389–20399 (2022). <https://doi.org/10.1021/acsnano.2c06362>
19. W. Li, H. Xu, H. Zhang, F. Wei, T. Zhang et al., Designing ternary hydrated eutectic electrolyte capable of four-electron conversion for advanced Zn–I_2_ full batteries. Energy Environ. Sci. **16**(10), 4502–4510 (2023). <https://doi.org/10.1039/d3ee01567j>
20. Z. Zhang, Y. Zhu, M. Yu, Y. Jiao, Y. Huang, Development of long lifespan high-energy aqueous organic||iodine rechargeable batteries. Nat. Commun. **13**(1), 6489 (2022). <https://doi.org/10.1038/s41467-022-34303-8>
21. Y. Zou, T. Liu, Q. Du, Y. Li, H. Yi et al., A four-electron Zn-I_2_ aqueous battery enabled by reversible I^-^/I^2^/I^+^ conversion. Nat. Commun. **12**(1), 170 (2021). <https://doi.org/10.1038/s41467-020-20331-9>
22. T. Liu, C. Lei, H. Wang, J. Li, P. Jiang et al., Aqueous electrolyte with weak hydrogen bonds for four-electron zinc-iodine battery operates in a wide temperature range. Adv. Mater. **36**(32), e2405473 (2024). <https://doi.org/10.1002/adma.202405473>
23. Y. Liu, L. Zhang, L. Liu, Q. Ma, R. Wang et al., All-climate energy-dense cascade aqueous Zn-I2 batteries enabled by a polycationic hydrogel electrolyte. Adv. Mater. 2415979 (2025). <https://doi.org/10.1002/adma.202415979>
24. S. Chen, Y. Ying, S. Wang, L. Ma, H. Huang et al., Solid interhalogen compounds with effective Br0 fixing for stable high-energy zinc batteries. Angew. Chem. Int. Ed. **62**(19), e202301467 (2023). <https://doi.org/10.1002/anie.202301467>
25. G. Liang, B. Liang, A. Chen, J. Zhu, Q. Li et al., Development of rechargeable high-energy hybrid zinc-iodine aqueous batteries exploiting reversible chlorine-based redox reaction. Nat. Commun. **14**(1), 1856 (2023). <https://doi.org/10.1038/s41467-023-37565-y>
26. H. Ye, J.J. Xu, Zinc ion conducting polymer electrolytes based on oligomeric polyether/PVDF-HFP blends. J. Power Sources **165**(2), 500–508 (2007). <https://doi.org/10.1016/j.jpowsour.2006.10.042>
27. J. Liu, Z. Khanam, R. Muchakayala, S. Song, Fabrication and characterization of Zn-ion-conducting solid polymer electrolyte films based on PVdF-HFP/Zn(Tf)_2_ complex system. J. Mater. Sci. Mater. Electron. **31**(8), 6160–6173 (2020). <https://doi.org/10.1007/s10854-020-03169-1>
28. C. Li, Z. Sun, T. Yang, L. Yu, N. Wei et al., Directly grown vertical graphene carpets as Janus separators toward stabilized Zn metal anodes. Adv. Mater. **32**(33), e2003425 (2020). <https://doi.org/10.1002/adma.202003425>
29. X. Zhang, J. Li, K. Qi, Y. Yang, D. Liu et al., An ion-sieving Janus separator toward planar electrodeposition for deeply rechargeable Zn-metal anodes. Adv. Mater. **34**(38), 2205175 (2022). <https://doi.org/10.1002/adma.202205175>
30. Z. Zheng, S. Guo, M. Yan, Y. Luo, F. Cao, A functional Janus Ag nanowires/bacterial cellulose separator for high-performance dendrite-free zinc anode under harsh conditions. Adv. Mater. **35**(47), 2304667 (2023). <https://doi.org/10.1002/adma.202304667>
31. Y. Kang, G. Chen, H. Hua, M. Zhang, J. Yang et al., A Janus separator based on cation exchange resin and Fe nanoparticles-decorated single-wall carbon nanotubes with triply synergistic effects for high-areal capacity Zn−I2 batteries. Angew. Chem. Int. Ed. **62**(22), e202300418 (2023). <https://doi.org/10.1002/anie.202300418>
32. K. Zhu, X. Niu, W. Xie, H. Yang, W. Jiang et al., An integrated Janus hydrogel with different hydrophilicities and gradient pore structures for high-performance zinc-ion batteries. Energy Environ. Sci. **17**(12), 4126–4136 (2024). <https://doi.org/10.1039/D4EE01018C>
33. S. Jin, Y. Deng, P. Chen, S. Hong, R. Garcia-Mendez et al., Solid-adsorbed polymer-electrolyte interphases for stabilizing metal anodes in aqueous Zn and non-aqueous Li batteries. Angew. Chem. Int. Ed. **62**(18), e202300823 (2023). <https://doi.org/10.1002/anie.202300823>
34. Y. Yan, S. Duan, B. Liu, S. Wu, Y. Alsaid et al., Tough hydrogel electrolytes for anti-freezing zinc-ion batteries. Adv. Mater. **35**(18), e2211673 (2023). <https://doi.org/10.1002/adma.202211673>
35. C. Li, W. Wang, J. Luo, W. Zhuang, J. Zhou et al., High-fluidity/high-strength dual-layer gel electrolytes enable ultra-flexible and dendrite-free fiber-shaped aqueous zinc metal battery. Adv. Mater. **36**(21), e2313772 (2024). <https://doi.org/10.1002/adma.202313772>
36. H. Zhang, X. Gan, Y. Yan, J. Zhou, A sustainable dual cross-linked cellulose hydrogel electrolyte for high-performance zinc-metal batteries. Nanomicro Lett. **16**(1), 106 (2024). <https://doi.org/10.1007/s40820-024-01329-0>
37. H. Lu, J. Hu, X. Wei, K. Zhang, X. Xiao et al., A recyclable biomass electrolyte towards green zinc-ion batteries. Nat. Commun. **14**(1), 4435 (2023). <https://doi.org/10.1038/s41467-023-40178-0>
38. F. Wang, J. Zhang, H. Lu, H. Zhu, Z. Chen et al., Production of gas-releasing electrolyte-replenishing Ah-scale zinc metal pouch cells with aqueous gel electrolyte. Nat. Commun. **14**(1), 4211 (2023). <https://doi.org/10.1038/s41467-023-39877-5>
39. H. Xia, G. Xu, X. Cao, C. Miao, H. Zhang et al., Single-ion-conducting hydrogel electrolytes based on slide-ring pseudo-polyrotaxane for ultralong-cycling flexible zinc-ion batteries. Adv. Mater. **35**(36), 2301996 (2023). <https://doi.org/10.1002/adma.202301996>
40. Z.-J. Chen, T.-Y. Shen, M.-H. Zhang, X. Xiao, H.-Q. Wang et al., Tough, anti-fatigue, self-adhesive, and anti-freezing hydrogel electrolytes for dendrite-free flexible zinc ion batteries and strain sensors. Adv. Funct. Mater. **34**(26), 2314864 (2024). <https://doi.org/10.1002/adfm.202314864>
41. H. Lu, J. Hu, L. Wang, J. Li, X. Ma et al., Multi-component crosslinked hydrogel electrolyte toward dendrite-free aqueous Zn ion batteries with high temperature adaptability. Adv. Funct. Mater. **32**(19), 2112540 (2022). <https://doi.org/10.1002/adfm.202112540>
